# Inflammasome NLRP3 activation induced by Convulxin, a C-type lectin-like isolated from *Crotalus durissus terrificus* snake venom

**DOI:** 10.1038/s41598-022-08735-7

**Published:** 2022-03-18

**Authors:** Cristina M. A. Rego, Aleff F. Francisco, Charles N. Boeno, Mauro V. Paloschi, Jéssica A. Lopes, Milena D. S. Silva, Hallison M. Santana, Suzanne N. Serrath, Jaína E. Rodrigues, Caleb T. L. Lemos, Ricardo S. S. Dutra, Jorddy N. da Cruz, Cleydson Breno R. dos Santos, Sulamita da S. Setúbal, Marcos R. M. Fontes, Andreimar M. Soares, Weverson L. Pires, Juliana P. Zuliani

**Affiliations:** 1 Laboratory of Cellular Immunology Applied to Health, Oswaldo Cruz Foundation, FIOCRUZ Rondônia, Porto Velho, RO Brazil; 2 Department of Physics and Biophysics, Institute of Biosciences, São Paulo State University, UNESP, Botucatu, SP Brazil; 3 Laboratory of Modeling and Computational Chemistry, Department of Biological and Health Sciences, Federal University of Amapá, Macapá, AP Brazil; 4 Center of Biomolecules Applied to Health (CEBio), Fundação Oswaldo Cruz, FIOCRUZ Rondônia, Porto Velho, RO Brazil; 5 Department of Medicine, Federal University of Rondônia, UNIR, Porto Velho, RO Brazil

**Keywords:** Immunology, Structural biology

## Abstract

Convulxin (CVX), a C-type lectin-like protein isolated from the venom of the snake species, *Crotalus durissus terrificus*, stimulates platelet aggregation by acting as a collagen receptor agonist for glycoprotein VI found in the platelets. The effect of CVX on platelets has been studied, but its effect on human peripheral blood mononuclear cells (PBMCs) remains unclear. Given the significance of PBMCs in inflammation, this study explored the effect of CVX on PBMCs, specifically regarding NLRP3 inflammasome activation by assessing cell viability, ability to induce cell proliferation, reactive oxygen species (ROS) and nitric oxide production, interleukin (IL)-2 and IL-10 secretion, NLRP3 complex activation, and the role of C-type lectin-like receptors (CTLRs) in these. CVX was not toxic to PBMCs at the investigated concentrations and did not increase PBMC growth or IL-2 release; however, CVX induced IL-10 release and ROS generation via monocyte activation. It also activated the NLRP3 complex, resulting in IL-1β induction. Furthermore, the interaction between CVX and Dectin-2, a CTLR, induced IL-10 production. CVX interaction with CTLR has been demonstrated by laminarin therapy. Because of the involvement of residues near the Dectin-2 carbohydrate-recognition site, the generation of ROS resulted in inflammasome activation and IL-1β secretion. Overall, this work helps elucidate the function of CVX in immune system cells.

## Introduction

*Crotalus durissus* is the main rattlesnake species distributed in South America and is also classified into subspecies. The venom composition of *Crotalus* subspecies varies, which may result in differences in its intensity and characteristics related to clinical manifestations in humans and in animal models^1–3^.

Previous studies have shown that *Crotalus* venoms stimulate lower levels of protective antibodies compared to other snake venoms, raising the hypothesis that the venom contains immunosuppressive component(s)^4,5^. Further, *Crotalus* venoms were demonstrated to be responsible for inducing anti-inflammatory, immunosuppressive, and analgesic effects in animal models^6–10^.

Convulxin (CVX), a C-type lectin-like venom component isolated from *Crotalus durissus terrificus* snake venom, possesses platelet aggregation activity and is of significant interest in toxinology because of its agonist effect on the glycoprotein VI (GPVI) receptor, the main signaling receptor for collagen^11,12^. CVX found in the venom of *C. durissus terrificus* is a C-type lectin-like protein with no carbohydrate affinity. It causes loss of balance, gastric abnormalities, seizures, and visual alterations *in viv*o and also induces platelet aggregation and cerebral ischemia^13^, in addition to its clinical manifestations. CVX is an octamer composed of four α and four β subunits, capable of grouping different receptors^14,15^. The protein is a disulfide-bonded heterodimer composed of two homologous subunits, CVXα (13.9 kDa) and CVXβ (12.6 kDa), which share substantial similarity with the carbohydrate recognition domain (CDR) of the C-type lectin family. CVX differs from other C-type lectins, because it lacks the consensus sequence for carbohydrate and Ca^2+^ binding, which is formed by alternative splicing in protein domains, resulting in loss of binding to Ca^2+^ due to the exchange of amino acids Lys45 and Lys130 with amino acids His45 and Lys122, respectively^16–19^. Despite its action on platelets, the effect of CVX on immune system cells remains unclear. Cells of the innate immune system express pattern recognition receptors (PRRs) that detect the basic components necessary for microbial engulfment, as well as sense danger signals against the cells. PRRs can detect ligands in the extracellular environment, whereas others, such as NOD-like receptors, offer immunological monitoring in the cytoplasm^20^.

Inflammasomes are multimeric complexes that develop in response to a wide range of physiological and pathological stimuli. The most widely studied inflammasome in innate immune cells is NLRP3 (Nucleotide and oligomerization domain, leucine-rich repeat-containing protein family, and pyrin-containing domain 3 receptor)^21^. These receptors can form inflammasomes and activate the cysteine protease, caspase-1, which catalyzes the cleavage of pro-interleukin-1β (pro-IL-1β) into mature IL-1β^22^. Thus, the current study was designed to assess the effects of CVX) on NLRP3 activation in peripheral blood mononuclear cells (PBMCs), as well as the potential C-type lectin-like receptors (CTLR) implicated in this process.

## Results

### CVX is not toxic to human PBMCs

**Figure 1:**
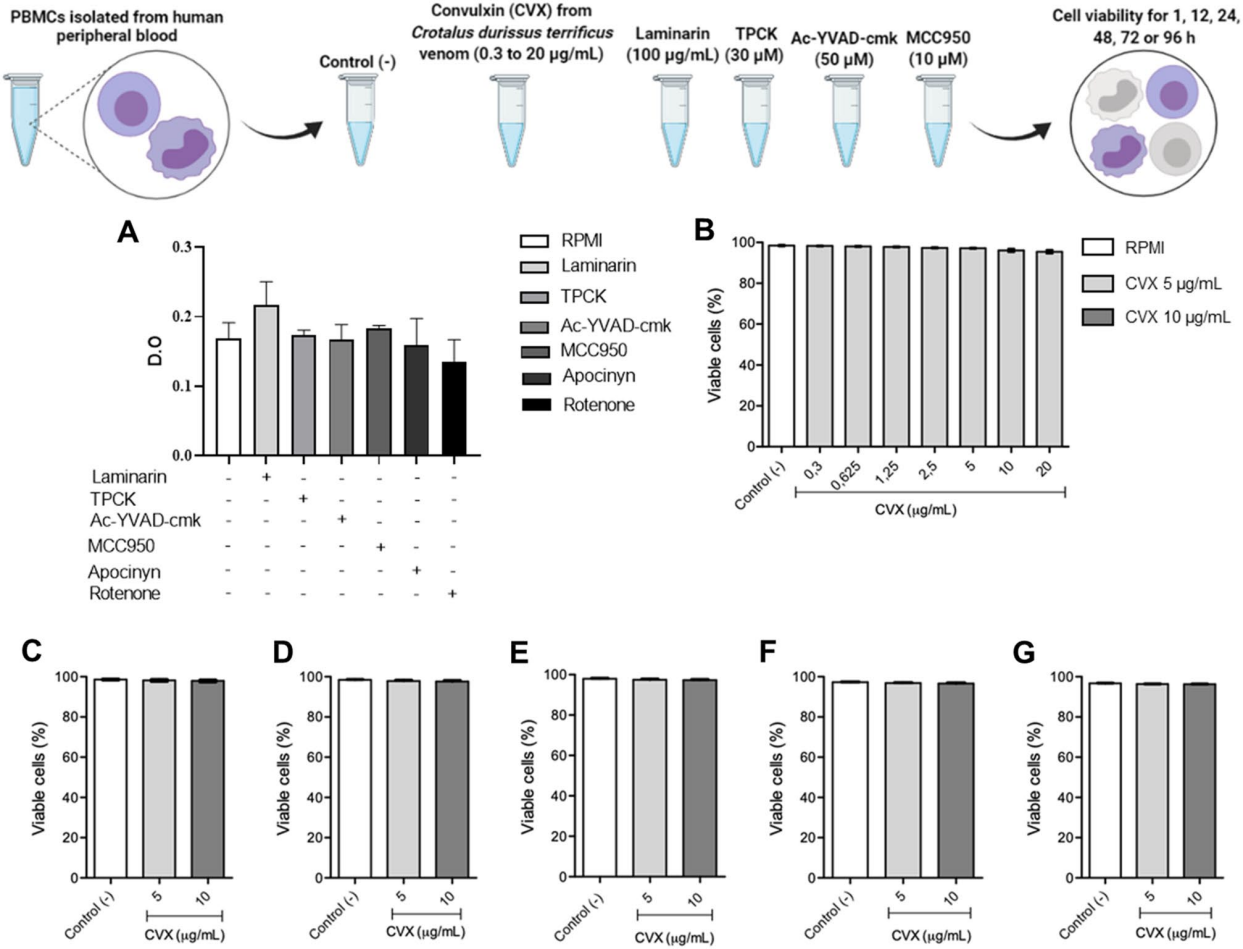
Cell viability of PBMCs in the presence of Convulxin (CVX). Human PBMCs isolated from leukocytes from healthy adult blood donors by a density gradient method viability in the absence or presence of inhibitors was measured by MTT metabolization method (**A**). Isolated 2 × 10^5^ PBMCs were incubated with RPMI (control) or with different concentrations of CVX (0.3 to 20 μg/mL; experimental group) for 1 h (**B**), or CVX (5 and 10 μg/mL; experimental group) for 12 (**C**), 24 (**D**), 48 (**E**), 72 (**F**), and 96 h (**G**). Viability was then assessed by PI and TO method in FACScan. The results were expressed in % and represent the mean ± S.E.M. from 4 to 5 donors.

To further investigate the effect of CVX on PBMCs function, we isolated these cells by density gradient, according to Pires et al.^23^. Cell viability was initially evaluated within 1 h at different concentrations (0.3, 0.625, 1.25, 2.5, 5, 10, and 20 μg/mL) of CVX for the definition of the concentration–response. Both 5 and 10 μg/mL concentrations were not toxic and were selected for the next experiments (Fig. 1B). After the concentration determination, cell viability was assessed at 12, 24, 48, 72, and 96 h using propidium iodide (PI) and thiazole orange (TO) staining and analysis by FACSCan. As can be seen in Fig. 1C–G, the incubation of PBMCs with RPMI (negative control) or CVX (5 or 10 μg/mL) at different times intervals did not affect cell viability.

To assess the cytotoxicity of the used inhibitors for cell signaling, the cell viability assay was performed using the 3-(4,5-dimethylthiazol-2yl)-2,5-diphenyl tetrazolium bromide (MTT) method. The cells were separated by density gradient, incubated with RPMI (negative control), laminarin (a β-glucan receptor ligand; 100 µg/mL), N-tosyl-l phenylalanine chloromethyl ketone (TPCK) (an inhibitor of NFκB Activation by Blocking Specific Cysteine Residues of IκB Kinase; NFκB inhibitor; 30 µM)^24^, Ac-YVAD-cmk (a selective, irreversible inhibitor of interleukin-1β converting enzyme ICE; caspase-1 inhibitor; 50 µM)^25^, MCC950 (a selective NOD-like receptor protein-3-NLRP3 inhibitor; 10 µM)^26^, Apocynin (a selective inhibitor of the phagocyte NADPH oxidase Nox2; 300 µM) and Rotenone (inhibitor of mitochondrial electron transport at nicotinamide adenine dinucleotide (NADH):ubiquinone oxidoreductase, mROS inhibitor; 10 µM) diluted in RPMI assay medium. The cell viability of PBMCs was tested after 12 h of incubation with the inhibitors utilized, and it was discovered that the inhibitors used had no effect on the viability of the PBMCs (Fig. 1A).

### CVX does not stimulate the proliferation of human PBMCs

**Figure 2:**
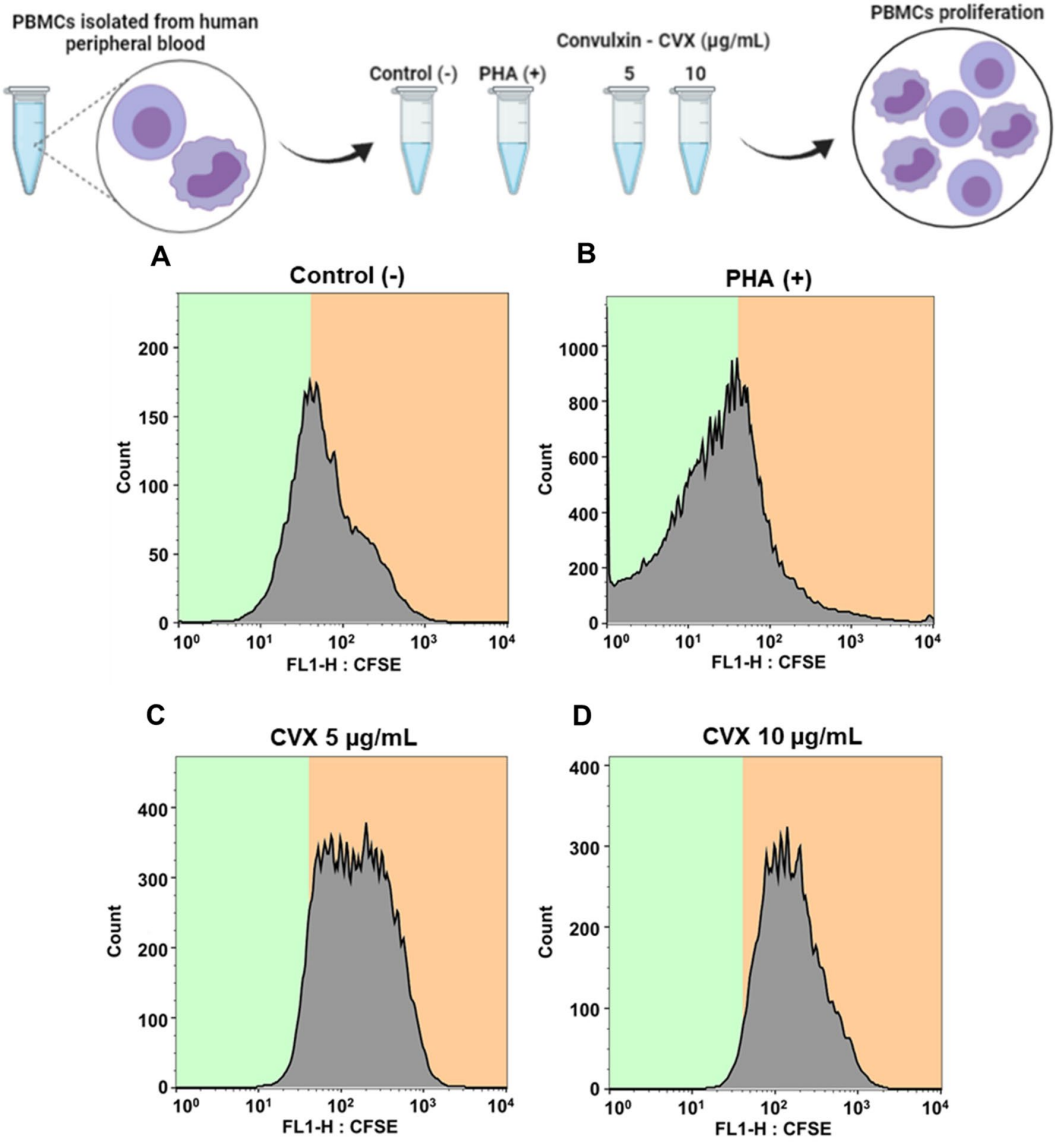
Cell proliferation of PBMCs in the presence of Convulxin. CFSE labelling of 2 × 10^5^ PBMCs proliferation followed by stimulation with RPMI (negative control) (**A**), PHA (5 µg/mL; positive control) (**B**) or CVX (5 and 10 μg/mL) (**C**,**D**) for 72 h and were analyzed in FACScan. The results were expressed as counts in histograms and represent the mean ± S.E.M of 3 donors.

To evaluate the effect of CVX on PBMCs proliferation the 5(6)-Carboxyfluorescein diacetate N-succinimidyl ester (CFSE) method was used. In Fig. 2, as can be seen, cells incubated with Phytohemagglutinin (PHA) (a positive control) induced a significant PBMCs proliferation compared to the cells incubated with RPMI (a negative control). However, CVX was not able to induce PBMCs proliferation at both concentrations used (5 and 10 μg/mL).

### CVX does not stimulate the secretion of Interleukin-2 (IL-2) but did stimulate the secretion of Interleukin-10 (IL-10) by human PBMCs

**Figure 3:**
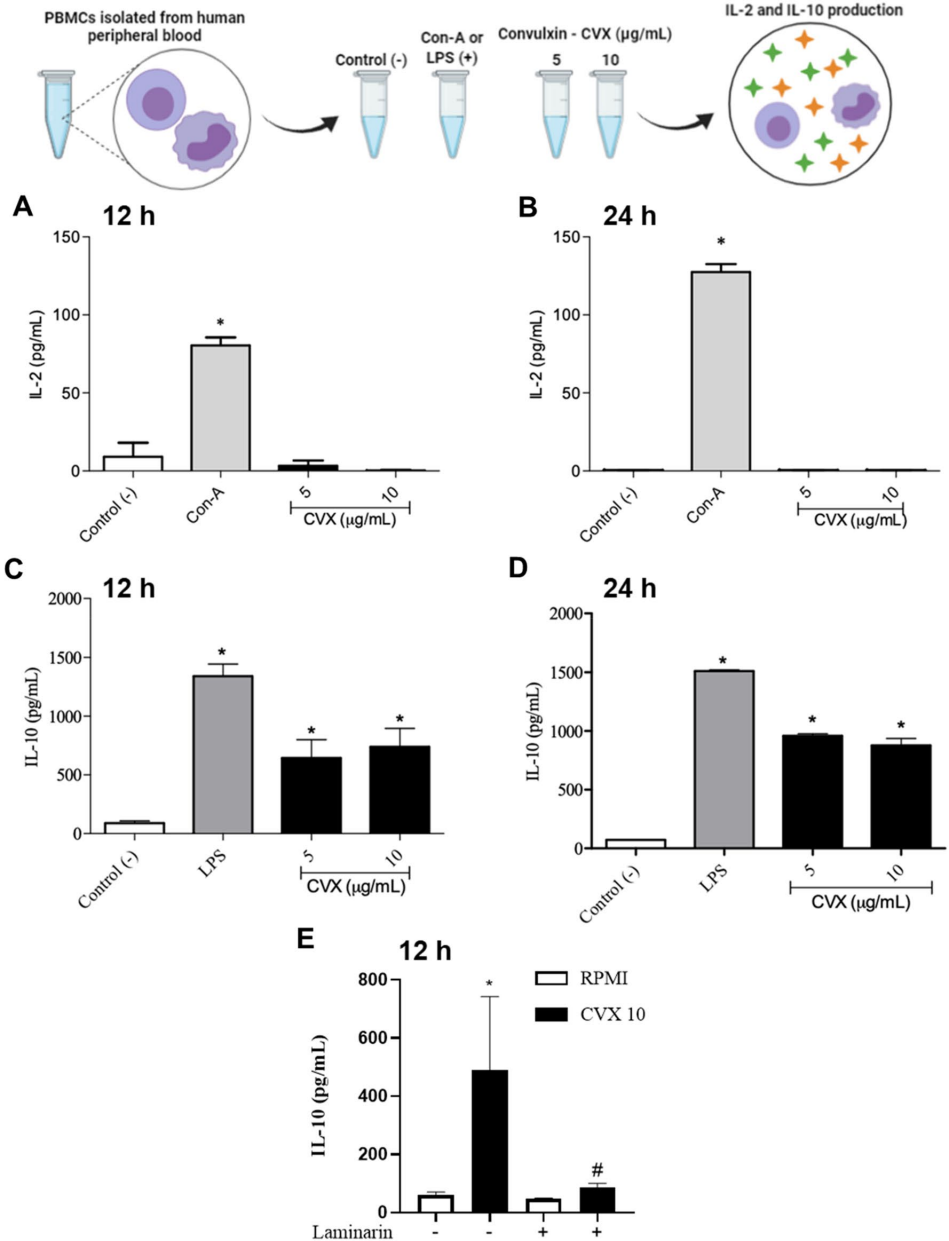
Interleukin-2 (IL-2) and Interleukin-10 (IL-10) production in PBMCs in the presence of Convulxin. Cytokines quantification in the supernatant of 2 × 10^5^ PBMCs by EIA followed by stimulation with RPMI (negative control), ConA (5 µg/mL; positive control), LPS (1 μg/mL; positive control) or CVX (5 and 10 μg/mL) for 12 and 24 h. IL-2 (**A**,**B**) and IL-10 (**C**,**D**) concentrations were quantitated by EIA assay in the supernatant collected after incubation with RPMI, ConA or LPS or CVX in spectrophotometer. IL-10 release was also quantified in PBMCs pre-treated with laminarin (100 µg/mL) for 30 min and after incubated with RPMI (negative control) or CVX (10 μg/mL) (**E**). The results were expressed as pg/mL of cytokines liberated and represent the mean ± S.E.M of 3 donors. ^*^P < 0.05 compared to negative control, #*P* < 0.05 compared to untreated PBMCs incubated with CVX (Data were presented with ANOVA followed by Tukey post-test).

In order to understand the effect of CVX on PBMCs proliferation, IL-2 and IL-10 production were evaluated. Figure 3 showed that PBMCs incubated with RPMI (a negative control) did not produce IL-2 at both times studied*.* In contrast*,* incubation of cells with Concanavalin A (Con-A), a known inducer of cell proliferation, significantly induced PBMCs to produce IL-2 at both times evaluated. Regarding CVX, at concentrations of 5 and 10 μg/mL, this lectin did not stimulate PBMCs to produce IL-2 suggesting that together with PBMCS proliferation assay, CVX does not have a role in this effect. IL-10 production was performed at 12 and 24 h of incubation with different stimuli. It was observed that PBMCs incubated with RPMI (a negative control) did not produce IL-10 at both times evaluated. On the other hand, incubation of cells with Lipopolysaccharide (LPS) (a positive control) significantly induced IL-10 production by PBMCs at both times evaluated. Similarly, CVX at concentrations of 5 and 10 μg/mL stimulated PBMCs to produce a significant amount of IL-10 at both studied periods (Fig. 3). According to the literature, the interaction of IL-10 with its receptor induces a signaling pathway via JAK/STAT culminating in transcription, production, and, IL-2 release^27^. Here, the possibility of CVX interaction with Dectin-2, a CTLR with the participation of IL-10 secreted was performed in PBMCs treated with laminarin, a β-glucan ligand for Dectin receptor. Results showed that PBMCs treated with laminarin blocked the IL-10 release induced by CVX (Fig. 3) demonstrating that CVX can interacts with Dectin-2, a CTLR resulting in an anti-inflammatory response.

### CVX does not stimulate the nitric oxide (NO) production by human PBMCs

**Figure 4:**
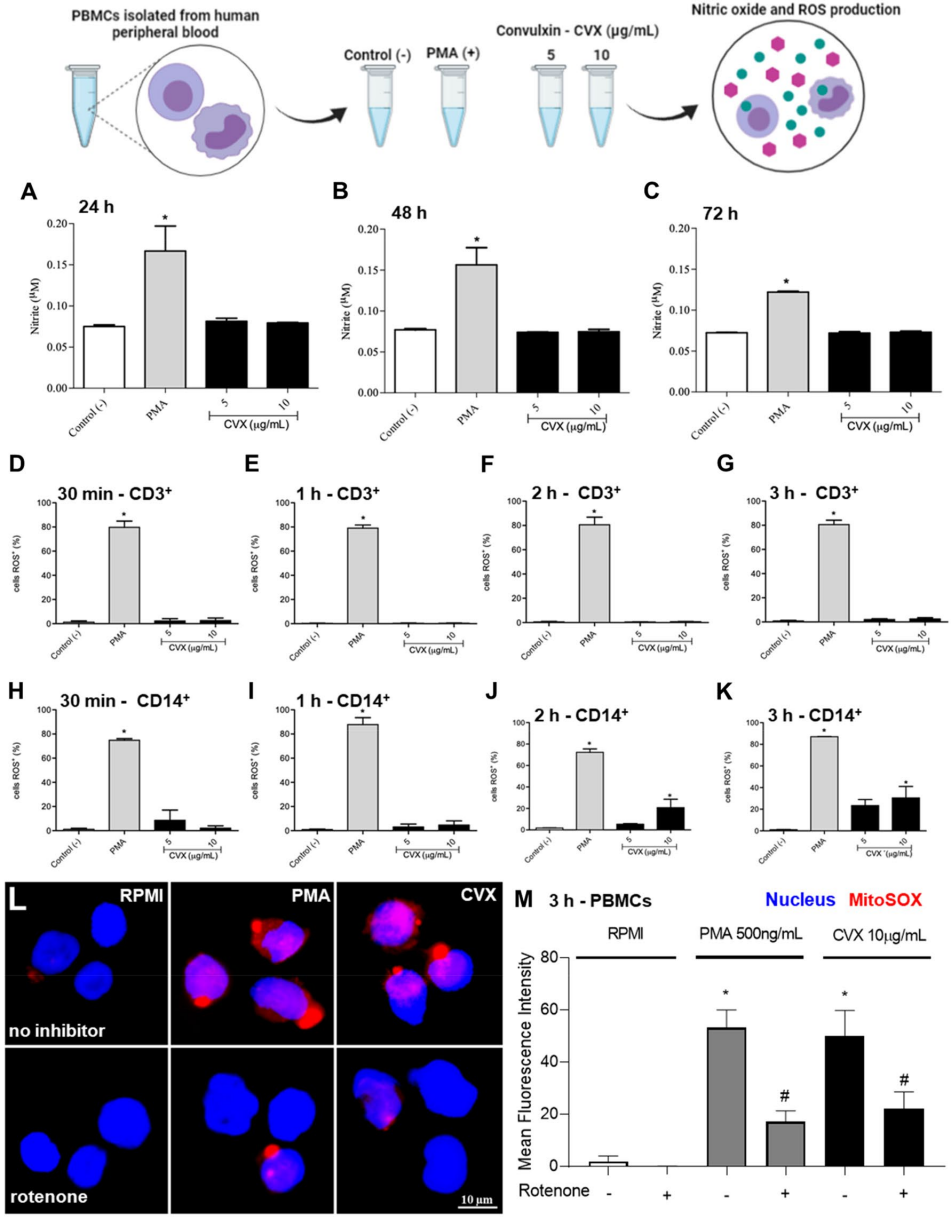
Nitrite and ROS production in PBMCs in the presence of Convulxin. NO quantification in the supernatant of 2 × 10^5^ PBMCs and ROS determination in 1 × 10^6^ CD^3+^ and CD^4+^ cells with DCFDA were quantified by flow cytometer followed by stimulation with RPMI without phenol red (negative control), PMA (500 ng/mL; positive control) or CVX (5 and 10 μg/mL) for selected time intervals. NO concentrations were quantitated by Griess method in supernatant collected after incubation with RPMI, LPS or CVX. ROS were determined in CD^3+^ and CD^14+^ gated populations following the addition of DCFDA (10 μM), and the DCF fluorescence was determined in FL1 channel in FACScan. The results were expressed as μM of NO liberated (**A**–**C**) and % of cell producing ROS and represent the mean ± S.E.M of 3 donors (**D**–**K**). ^*^P < 0.05 compared to negative control (Data were presented with ANOVA followed by Tukey post-test). The images were collected using constant automatic gain among the samples to quantify the differences in absolute levels of fluorescence intensity of different conditions in 100 × magnification oil immersion objective. Figure representative of one experiment of three independent experiments (**L**,**M**). Analysis of the mean fluorescence intensity of ROS immunofluorescence was performed using 10 cells in field of view of each condition collected impartially.

In order to continue to investigate the effects of CVX, the ability of this lectin to induce NO production by human PBMCs were conducted. The cells were incubated with RPMI (a negative control), Phorbol 12-myristate 13-acetate (PMA) (a positive control), or CVX (5 and 10 µg/mL) for different time intervals. As shown in Fig. 4A–C, the incubation of PBMCs with PMA induced a significant increase in NO production compared to control cells. On the other hand, CVX at concentrations of 5 and 10 µg/mL did not induce NO production compared to control cells, which suggests that this lectin does not stimulate PBMCs to produce nitric oxide.

### CVX does not stimulate the production of reactive oxygen species (ROS) by human CD^3+^, but it does by CD^14+^

ROS were measured by spectrophotometry and flow cytometry methods. It was observed that when evaluated by spectrophotometry, cells incubated with PMA (a positive control) showed a significant increase in ROS production at 30 min and 1 h, returning to basal levels at 2 and 3 h. The cells incubated with CVX at concentrations of 5 and 10 μg/mL did not induce ROS production at any time evaluated. The same observation was achieved with cells incubated with RPMI (a negative control) (Fig. 4).

Flow cytometry analysis demonstrated that PBMCs incubated with RPMI (a negative control) and gated in CD^3+^ (Fig. 4D–G) and CD^14+^ populations (Fig. 4H–J) showed that lymphocytes and monocytes, respectively, did not produce ROS at all period studied. However, the incubation of PBMCs with PMA (a positive control) showed that the gated CD^3+^ and CD^14+^ populations induced a significant ROS production at all periods studied. This effect was not observed when cells were incubated with CVX for CD^3+^ population but monocytes, the CD^14+^ population, induced a significant ROS production after 2 and 3 h incubation with the lectin.

To assess the production of mitochondrial ROS by PBMCS, the cells were subjected to pharmacological treatment with Rotenone (10 µM), followed by labelling the cells by the MitoSOX ™ Red Mitochondrial Superoxide Indicator method (Fig. 4L,M). The results showed that CVX induced mitochondrial ROS and this effect was decreased in the presence of the inhibitor, indicating that signals for complex activation by mitochondrial ROS were suppressed.

### CVX activates the NLRP3 inflammasome complex in PBMCs

**Figure 5:**
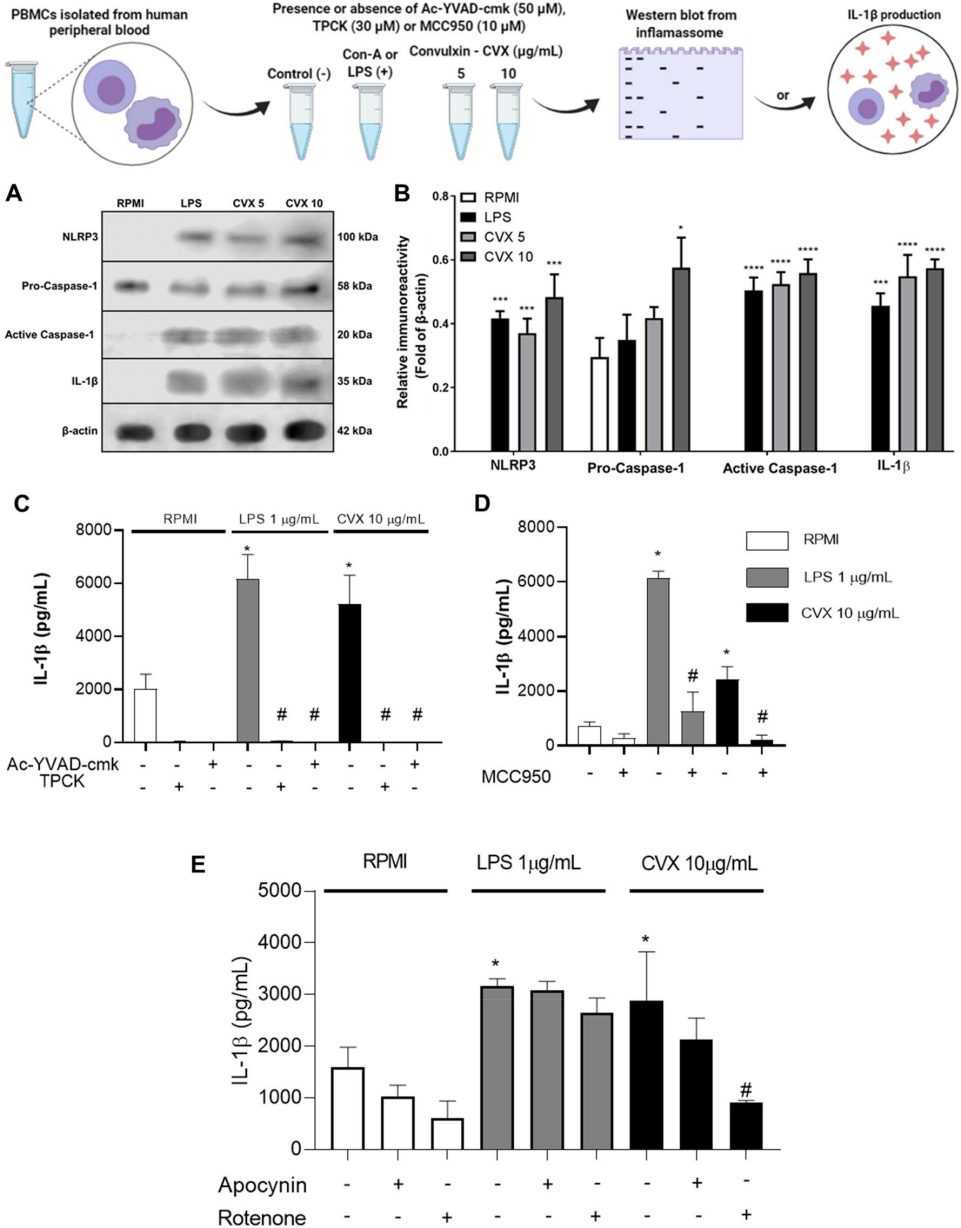
Protein expression of inflammasome NLRP3 in PBMCs in the presence of Convulxin. Western blot of NLRP3, Pro-Caspase-1, Active Caspase-1, IL-1β, and β-actin (control) using 1 × 10^7^ PBMCs stimulated RPMI (negative control), LPS (1 μg/mL; positive control) or CVX (5 and 10 μg/mL) for 3 h. (**A**) Figure representative of one experiment of three independent experiments. Relative immunoreactivity analysis (fold of β-actin) of the western blots from NLRP3, Pro-Caspase-1, Active Caspase-1, IL-1β (**B**). IL-1β release was also quantified in PBMCs pretreated with N-tosyl-l-phenylalanine chloromethyl ketone (TPCK) (30 µM), Ac-YVAD-cmk (50 µM) (**C**), MCC950 (10 µM) (**D**) for 30 min and after incubated with RPMI (negative control) or CVX (10 µg/mL). IL-1β was also quantified in PBMCs pretreated with Apocynin (300 µM) and Rotenone (10 µM) for 30 min and after incubated with RPMI (negative control) or CVX (10 µg/mL) (**E**). Results were expressed in pg/mL of released cytokines and represent the mean ± S.E.M of 3 donors. Values are mean S.E.M. from 3 donors. *P < 0.05, **P < 0.01, ***P < 0.001, ****P < 0.0001 compared to negative control (data were presented with ANOVA followed by Dunnett post-test).

After 3 h of PBMCs incubation with RPMI, LPS or CVX, the NLRP3, cleaved caspase-1, and IL-1β protein expression were investigated. It was observed that both LPS (a positive control) and CVX at concentrations of 5 and 10 µg/mL induced NLRP3, cleaved caspase-1, and IL-1β protein expression. This aspect was not observed in the PBMCs incubated with RPMI (a negative control) showing that CVX induces the activation of NLRP3 inflammasome complex (Fig. 5A,B).

### CVX stimulates PBMCs to secrete interleukin-1 (IL-1)

In order to continue understanding the effect of CVX on the activation of the inflammasome complex in PBMCs, the production of IL-1β a product of the formation of the NLRP3 inflammasome complex was evaluated. This cytokine was induced by the positive control (LPS) and by the CVX at a concentration of 10 µg/mL. Therefore, the production of IL-1β under the action of CVX was performed in the absence or the presence of N-tosyl-L phenylalanine chloromethyl ketone (30 µM), Ac-YVAD-cmk (50 µM), or MCC950 (10 µM). The results showed that the release of IL-1β induced by CVX was inhibited in the presence of studied inhibitors (Fig. 5C,D) confirming that CVX induces the activation of NLRP3 inflammasome complex.

To assess the production of ROS as signaling mechanism for the activation of the inflammasome complex in the PBMCS, IL-1β was produced in the absence or presence of Apocynin (300 µM) or Rotenone (10 µM) (Fig. 5E). The results demonstrated that the release of cytokine via CVX was reduced, indicating that signals for the complex’s activation by mitochondrial ROS was inhibited.

### In silico, CVX interacts with Dectin-2, a CTLR

The interaction mechanism between CVX and CTLRs proposed herein was evaluated through docking and molecular dynamics (MD) simulations. Initially, CVX (αβ)_4_ tetramer was tested via protein–protein docking against CTLRs targets (CLEC4E and Dectin-2). Subsequently, CVX/CTLR complexes stability were evaluated with molecular dynamics.

**Figure 6:**
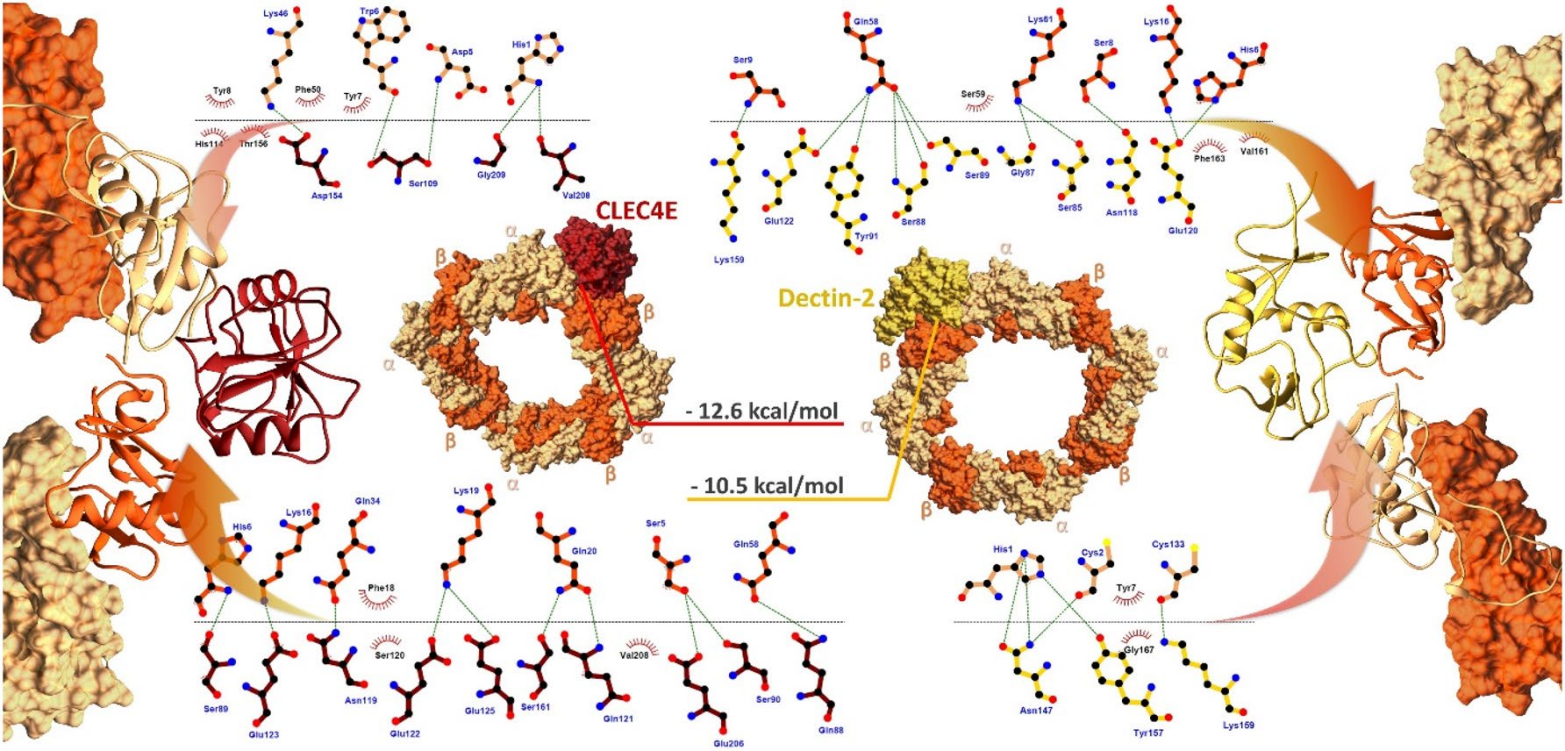
Complexes formed in the interaction of CVX (β subunits in orange and α subunits in sandy brown) with ectodomains of C-type Lectins receptors CLEC4E (red) and Dectin-2 (yellow). The complexes displayed above are the extracted central structures from the largest cluster of each respective MD trajectory, both structures are approximations of the most predominant conformations assumed by these proteins during 10 ns of simulation. The ΔG in kcal/mol of each interaction interface is highlighted in lines colored according to its complex of origin. Residues coordinating the interaction interfaces are highlighted on the interaction maps with hydrogen bonds depicted in green dashed lines and hydrophobic interactions highlighted in red protrusions.

**Figure 7:**
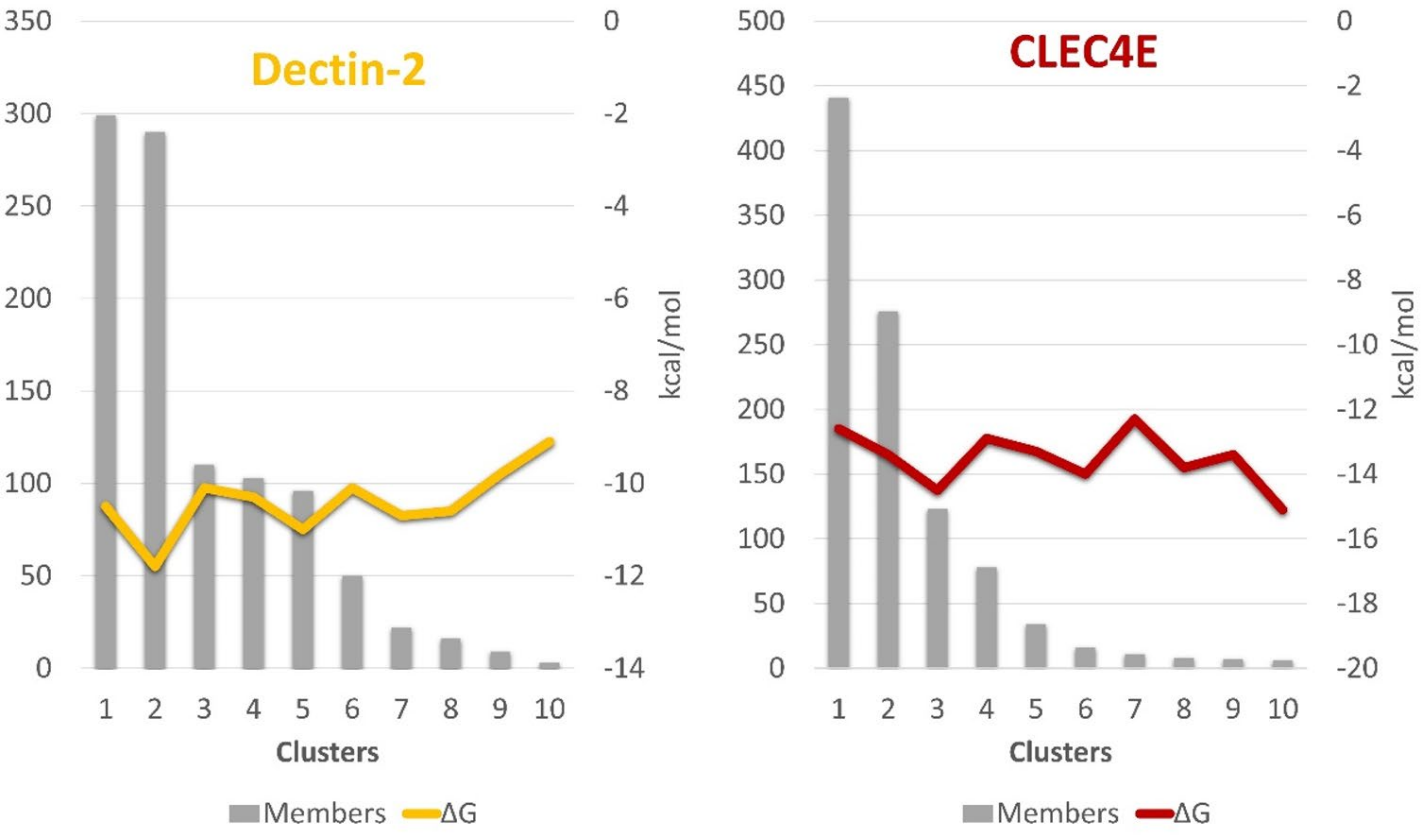
Molecular dynamics strategies of CVX/CLEC4E and CVX/Dectin-2 simulations. The graphs above exhibit the 10 most populated clusters (grey bars) extracted from the MD trajectories of CVX/CLEC4E and CVX/Dectin-2 simulations, relating the number of members per cluster and the variation in binding energy among the representants (central structure) of each cluster (red and yellow lines).

**Figure 8:**
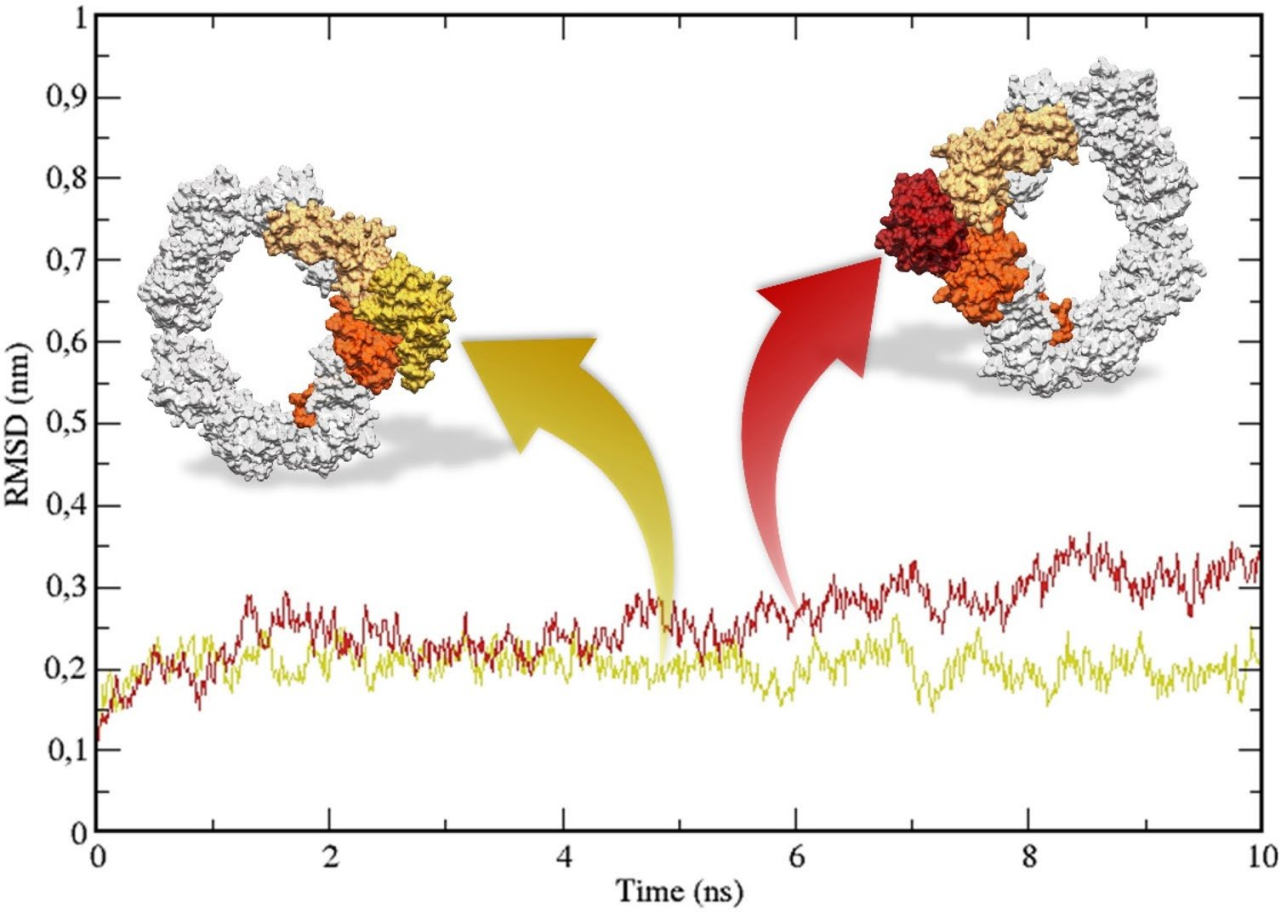
Backbone RMSD measures from the trajectories of CVX/CLEC4E (red) and CVX/Dectin-2 (yellow) simulations. To avoid any masking effect generated by the hole CVX (αβ)_4_ tetramer the RMSD was measured only from the main interacting parties, as highlighted in the image above.

The CVX/CLEC4E complex (Fig. 6) showed reasonable stability, evidenced by the simply root-mean-square deviation (RMSD) measurements (Fig. 7) of the CLEC4E and the CVX αβ subunits integrating the interaction interface. Additionally, the analysis of the residues that coordinate the interaction interface suggests the participation of CLEC4E residues from Ca^2+^ binding site (N119, Q206, Q123)^28^. CVX/CLEC4E clustering study revealed a favorable binding energy pattern throughout the entire simulation. Similarly, the CVX/Dectin-2 clustering also showed favorable binding energies (Fig. 8), with a slightly decrease in affinity when compared with CVX/CLEC4E. Nevertheless, the CVX/Dectin-2 appears to be more stable than the CVX/CLEC4E in the RMSD evaluation.

## Discussion

Along with its affinity for the collagen receptor glycoprotein VI (GPVI) in platelets, CVX exhibits platelet aggregation activity. CVX serves as an affinity ligand in the purification and sequencing of GPVI receptor, and its contact with platelets is mediated by processes previously reported in T and B lymphocytes^11,29^. Protein interactions with CTLR can induce three distinct cellular responses: production and release of IL-2, a cytokine responsible for lymphocyte proliferation; activation of NF-κB, which leads to the production of various cytokines and chemokines; and activation of the inflammasome complex, which leads to the release of IL-1β^30,31^.

First, the effect of CVX on PBMC viability was studied. As cell viability tests measure the metabolically active cells present in cultures, the concentration–response and time-course experiments revealed that CVX did not affect the viability of human PBMCs. Consistent with these observations in PBMCs, recent research has shown that CVX does not cause toxicity in smooth muscle cells of the human coronary artery (HCASMC) in cell viability tests after 18 h of incubation at a concentration of 0.24 M^32^.

Lymphocyte proliferation assays were performed to investigate the potential effect of CVX on PBMC activation, and we found that CVX did not promote lymphocyte proliferation at the concentrations tested (5 and 10 g/mL). The synthesis and release of IL-2, a cytokine responsible for lymphocyte proliferation, may be a possible mechanism underlying this effect. Thus, after quantifying IL-2, the results indicated that CVX did not stimulate the production of this cytokine by PBMCs at the two concentrations tested (5 and 10 g/mL) and after 12 and 24 h of incubation. As IL-2 is mitogenic for T cells, these findings support the results of the cell proliferation experiment. The interaction of C-type lectin-like protein with CTLR results in IL-2 production and release, with subsequent signaling mediated by mitogen-activated protein kinase (MAPK)^31^; therefore, it can be concluded that CVX does not activate this signaling cascade and thus cannot induce PBMC proliferation.

Shih et al.^32^ obtained comparable results using the MTT assay to assess the mitogenic activity of CVX in HCASMCs. Their results revealed that at the concentrations employed (0.06–0.6 mM), CVX inhibited cell growth in a dose-dependent manner compared to the controls. The inhibitory effect of CVX on HCASMC proliferation was found to be mediated by αvβ3 integrin. Furthermore, they demonstrated that the WAD tripeptide (similar to RGD present in disintegrins) found in the CVX-chain is responsible for influencing the activities mediated by αvβ3 integrin.

Therefore, CVX is hypothesized to be a C-type lectin-like protein with no mitogenic action in lymphocytes. Another theory is that CVX induces the synthesis of IL-10, a cytokine with anti-inflammatory properties, and therefore acts as a negative modulator of cell activation^30,33^. Therefore, we assessed the effect of CVX on IL-10 production in PBMCs and found that CVX stimulated the release of this cytokine at both concentrations and time intervals.

Laminarin, a Dectin ligand, was used to examine the receptor participation and thus to assess cell signaling for confirming the pathway that resulted in IL-10 generation. According to these findings, CVX did not promote IL-10 production in the presence of laminarin, indicating that interaction with Dectin-2, a CTLR, resulted in an anti-inflammatory response.

Human IL-10 inhibits the synthesis of pro-inflammatory cytokines such as IL-1, IL-2, IFN-γ, IL-4, and IL-5 at the transcriptional level. Notably, several cell types including monocytes, macrophages, dendritic cells, and lymphocytes, are capable of producing IL-10. Among its many functions, IL-10 has the capacity to suppress the proliferative responses of antigen-specific T cells. When triggered by stimuli, IL-10 diminishes T-lymphocyte responses by reducing the expression of monocyte major histocompatibility complex (MHC) II^34–38^.

The intracellular signaling that culminates in IL-10 production is independent of NF-κB; IL-10 binding to IL-10 receptor (IL-10R) activates the JAK-STAT signaling pathway, specifically Jak-1 (associated with the chain of the receptor) and Tyk2 (associated with the chain of the receptor), and induces Stat1, Stat3, and, in some cell models, Stat5^39^.

According to Shih et al.^32^, NF-κB activation does not appear to be involved in the signaling events for the expression of IL-8 and the growth-stimulating activity generated by CVX in HCASMC cells, thus explaining the negative regulation of these cytokines. The present results indicate that IL-10 generated by PBMCs under the influence of CVX suppressed IL-2 production, and thus suppressed cell proliferation.

Nitric oxide (NO) is another essential mediator of the inflammatory response. NO detection in the cell supernatant revealed that CVX did not cause PBMCs to generate NO; similar results were observed in experiments with another lectin. Pires et al.^23^ demonstrated that BjcuL, a C-type lectin isolated from *Bothrops jararacussu* venom, was unable to activate PBMCs and release NO within 96 h of incubation.

As NO is a cytotoxic and cytostatic molecule generated by phagocytic cells, it is hypothesized that the immunomodulatory function of IL-10 may block the effect of NO on PBMCs. Literature also shows that NO has an anti-proliferative effect on T cells and that this effect is mostly mediated by NO action donors and their role in regulating the production of cytokines such as IL-10^40–42^.

As seen in Fig. 10, CVX may also interact with CTLR, causing ROS generation and as a result, inflammasome activation. Spectrophotometry and flow cytometry were used to assess ROS generation in the PBMCs activated by CVX. When PBMCs were incubated with CVX, the spectrophotometry results indicated that ROS generation was similar to that at the baseline. However, immunophenotyping showed that, upon CVX exposure, the monocyte population (in a smaller proportion than lymphocytes) produced considerably more ROS compared to control cells after 2 and 3 h of incubation.

According to previous studies, one of the earliest triggers of NLRP3 inflammasome activation is ROS production, which occurs in the prolonged presence of damaged mitochondria. However, the means by which the NLRP3 inflammasome recognizes the synthesis of these reactive species remain unknown^43,44^. Thus, based on protein expression investigations of NLRP3 inflammasome components, CVX may activate this complex in PBMCs during the first 3 h of exposure.

Activation of the inflammasome complex results in maturation of the cytokine IL-1β via caspase-1. IL-1β production in PBMCs was thus assessed in the presence of CVX. CVX increased the secretion of this pro-inflammatory cytokine after 3 h of incubation, indicating activation of the inflammasome complex. To confirm this effect, PBMCs were treated with inhibitors of Caspase-1 and NLRP3 before stimulation with CVX. Induction of IL-1β synthesis by CVX was reduced by both inhibitors tested, supporting the previous findings demonstrating the role of CVX in NLRP3 inflammasome complex activation through protein expression and IL-1β production (Fig. 5).

To test ROS as an inflammasome complex activator, IL-1β production in PBMCs was measured in the presence of NADPH oxidase and mitochondrial ROS inhibitors, followed by CVX activation. The results showed that in the presence of CVX, ROS inhibition via mitochondria decreased the production of IL-1β by PBMCs (Fig. 5).

Zhou et al.^43^ investigated NLRP3 inflammasome activation by mitochondrial ROS signaling, which supports our findings. The production of mROS activated the NLRP3 inflammasome, and macrophages treated with NLRP3 activators were activated by recruiting ASC adaptor. This study establishes a connection between inflammasomes and mitochondrial function.

The use of rotenone as a mitochondrial ROS activator is known in the literature. However, experimental data demonstrated that this compound was able to inhibit mROS production in the presence of CVX and PMA (Fig. 4M). Corroborating our experimental findings, Pontes et al.^45^, evaluated the inhibition of mitochondrial ROS in neutrophils with the use of rotenone in the presence of Cr-LAAO. Data from Mills et al.^46^, also demonstrated the use of rotenone as an inhibitor in macrophages. The authors' experimental data demonstrated that rotenone inhibited mROS production in neutrophils and macrophages, confirming our experimental data.

In response to different pattern recognition receptor (PRR) ligands and cytokines, NF-κB functions as a key mediator of the NLRP3 inflammasome initiation signal, promoting the transcriptional production of NLRP3 and pro-IL-1β^47^. To determine the role of NF-κB in the activation of the inflammasome complex by CVX, PBMCs were cultured with an NF-κB inhibitor before being stimulated with CVX, and IL-1β production was measured. Our findings show that NF-κB is implicated in the ability of CVX to activate the inflammasome complex in PBMCs, resulting in the generation of IL-1β (Fig. 10).

The findings reported herein revealed that CVX is not toxic to human PBMCs. This C-type lectin-like protein has no mitogenic action on PBMCs and does not stimulate the generation of IL-2 or NO. However, CVX promoted the generation of mROS by monocytes, which prompted signaling for the activation of the inflammasome complex, resulting in the release of IL-1β, a pro-inflammatory cytokine. Thus, the collected data set led us to hypothesize that CVX can interact with CTLR, causing ROS generation, NF-κB, and inflammasome activation, and, as a result, IL-1β production, in addition to playing a role in the immunomodulation of PBMCs via IL-10 production (Fig. 10).

An *in-silico* investigation was carried to explore the interactions between CVX and CTLRs, seeking to evaluate this mechanism at the molecular level. As a result, two CTLR ectodomains were selected (CLEC4E and Dectin-2). These specific receptors were chosen based on the availability of crystallographic data, the cell types in which they are expressed, and the relationship between the in vitro discoveries reported herein and the consequences induced by activation upon contact with these receptors^48^.

CLEC4E is a type II transmembrane receptor that activates FcγRs in macrophages, dendritic cells, and monocytes. Binding events to this receptor cause the tyrosine-based immunoreceptor (ITAM) in the FcγR to be phosphorylated, resulting in the production of TNF and IL-6^28^. The predicted coupling mode in which the CVX binds to the CLEC4E ectodomain involves Ca^2+^ binding site residues of this receptor and a consistent binding free energy for the most predominant conformations observed in the MD simulation, indicating that this interaction might occur in the physiological scenario.

Dectin-2 is expressed on monocytes and macrophages and has a CRD (C-type carbohydrate-recognition domain) connected to its transmembrane region. In addition, the Dectin-2 surface structure is attached to FcγR, which mediates the connection with Syk kinase and produces cytokine secretion signaling^49^. Thus, the complex anticipated for the interaction between CVX and Dectin-2 points to an event that can elicit a response, especially given the involvement of residues near the Dectin-2 carbohydrate-recognition site.

**Figure 9:**
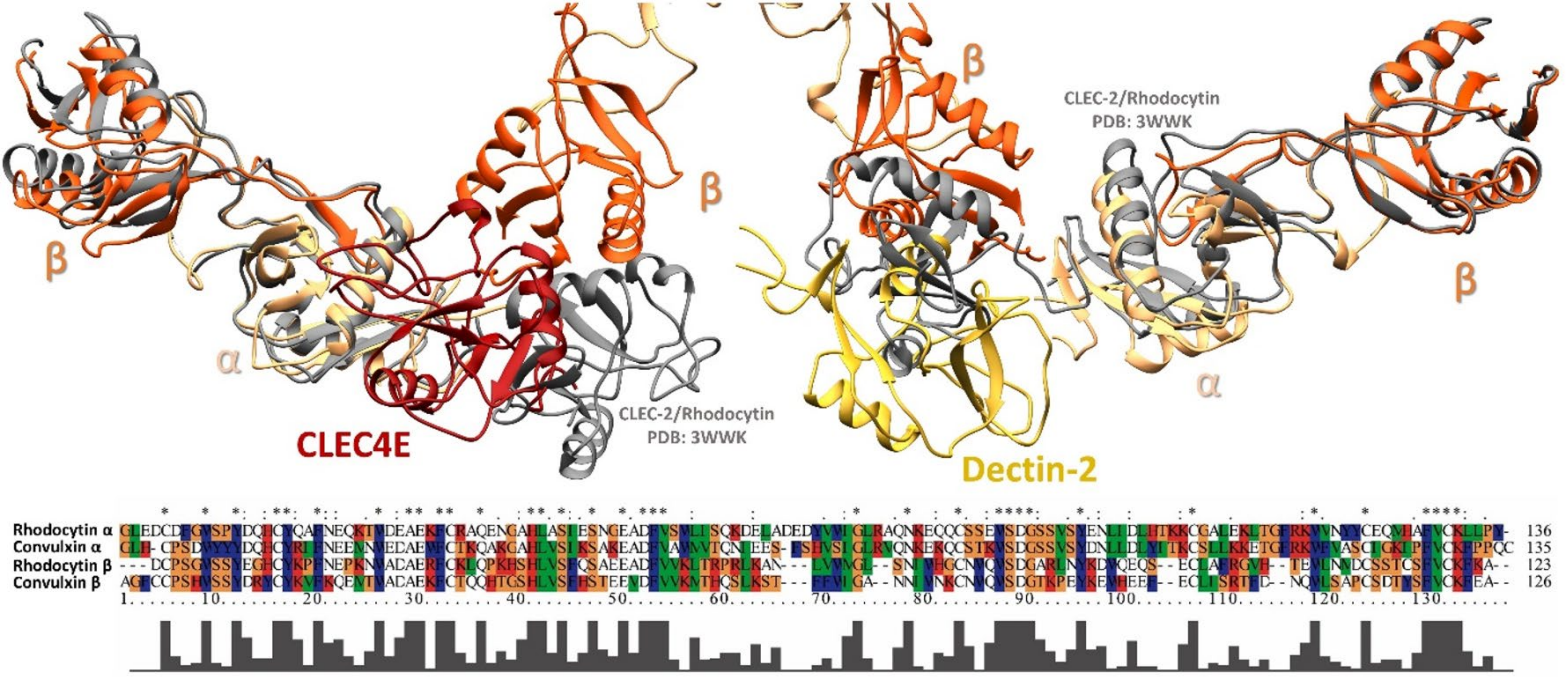
Superposition of Rhodocytin in complex with CLEC-2 (gray) crystal structure (PDB: 3WWK) and the complexes predicted for the interactions between CVX (β subunits in orange and α subunits in sandy brown) and the CTLRs CLEC4E (red) and Dectin-2 (yellow).

Even though the CVX canonical binding site lies at the concave surface of the dimers, CVX displayed an unanticipated binding pattern when interacting with the CTLRs^50^. The docking experiments indicated that CTLRs binding site is positioned at the interface between CVX two dimers (Fig. 6), with both complexes exhibiting a similar binding energy pattern^15^. Although surprising, this behavior has been previously documented in the literature and is consistent with crystallographic investigations conducted with *Calloselasma rhodostoma* rhodocetin and the CTLR CLEC-2^51^. Furthermore, the superposition of the crystal structures of rhodocetin complexed with CLEC-2 (PDB: 3WWK) and the CVX/CLEC4E and Dectin-2 complexes (Fig. 9) demonstrates the high degree of similarity shared by these SNACLEC, further supporting the *in-silico* interactions.

Overall, the in vitro and in silico results found in this study sustain the possibility that interaction with CTLRs may be the mechanism through which the CVX triggers the IL-10 as mentioned earlier and mROS production along with the inflammasome NLRP3 activation and a cascade of effects that follows this event culminating in IL-1β release.

## Methods

### Chemicals and reagents

Lipopolysaccharides from *Escherichia coli* O111:B4 (LPS), l-glutamine, gentamicin, concanavalin A (Con-A), phorbol myristate acetate (PMA), Histopaque 1077, DMSO, carboxyfluorescein diacetate succinimidyl ester (CFDA-SE), RPMI-1640, l-glutamine, 3,3′,5,5′-Tetramethylbenzidine (TMB), Bovine Serum Albumin (BSA), anti-rabbit horseradish peroxidase, anti-β-actin (A1978-200UL) and 3,3′diaminobenzidine (DAB), N-tosyl-l-phenylalanine chloromethyl ketone (TPCK), Ac-YVAD-cmk, Apocynin and Rotenone were purchased from Sigma Aldrich (MO, USA). MCC950 was purchased from InvivoGen (France). Anti-caspase-1(AG-20B-0044-C100) and anti-NLRP3 (AG-20B-0014) were from Adipogen Life Sciences (San Diego, CA, USA). Goat anti-mouse antibody conjugated to horseradish peroxidase (A90-116P) was purchased from Bethyl Laboratories (Montgomery, TX, USA). Anti-IL-1β (af-401-NA) was from R&D Systems. Fetal bovine serum (FBS) was obtained from Cultilab (São Paulo, Brazil). Cell Viability Kit, Human IL-10 ELISA, Human IL-2 ELISA, Human IL-1β ELISA, PE anti-human CD14, and PerCP anti-human CD3 were purchased from BD Pharmingen (CA, USA). 3-4,5 dimethylthiazol-2, 5 diphenyltetrazolium bromide (MTT), Pierce™ Chromogenic Endotoxin Quant Kit and MitoSOX ™ were obtained from Thermo Fisher Scientific (MA, USA). 2′,7′- dichlorodihydrofluorescein diacetate (DCFDA) was purchased from Molecular Probes (OR, USA). PVDF membrane and all salts and reagents used obtained from Merck Millipore (Darmstadt, Germany) with low endotoxin or endotoxin-free grades.

### Convulxin (CVX)

Dr. Andreimar M. Soares of the Centro de Estudos de Biomoléculas Aplicadas a Sade (CEBio)—FIOCRUZ-Rondonia provided the CVX, a C-type lectin-like identified from *Crotalus durissus terrificus* venom. CVX was purified using a SuperdexTM 75 column, and the fraction containing the lectin was subjected to a reverse-phage column for the second chromatographic stage^13^.

### Endotoxin quantification

For this assay, the Pierce™ Chromogenic Endotoxin Quant Kit was used. The plate was pre-warmed to 37 °C, followed by the addition of 50 µL of CVX and standards. Then, 50 µL of lysate was added, and incubated at 37 °C for 30 min. After 30 min, 100 µL of chromogenic substrate was added, and incubated at 37 °C for 6 min. After the reaction, the stopping solution was added. Absorbance was measured spectrophotometrically (Bio-Tek Synergy HT Multi-Detection, Winooski, VT) at 405 nm. Data were expressed in EU/mL. 0.5 EU/mL of endotoxin was detected in the CVX preparation. The sample used is within the acceptable threshold of 1 EU/mL according to Pinto et al.^52^.

### Ethical statement

The Brazilian Institutional Review Board of the Centre for Research in Tropical Medicine (CEPEM-RO) authorized this study under protocol number CAAE 71745917.1.0000.0011. All subjects were adults who completed a Statement of Informed Consent. All tests were carried out in compliance with the applicable rules and regulations.

### Isolation of human peripheral blood mononuclear cells (PBMC)

Blood was donated by healthy volunteers who had not used any medication in the last 48 h. Blood was collected in vacuum tubes containing heparin. PBMCs were isolated by a density gradient method as described by Pires et al.^23^ using Histopaque 1077 according to the manufacturer’s instructions. Briefly, the blood was layered on Histopaque at a 1:1 ratio and subjected to centrifugation at 400×*g* for 30 min. The white layer representing PBMCs was gently aspirated and aseptically transferred into sterile centrifuge tubes. After centrifugation, the cell suspension containing the PBMCs was washed 3 times with PBS and cultured in sterile RPMI assay medium [RPMI-1640 medium supplemented with 100 µg/mL of gentamicin, 2 mM of l-glutamine and 10% fetal bovine serum (FBS)]. Aliquots of the isolated PBMCs were used to determine the total number of cells in a Neubauer’s chamber following cell staining (1:20, v/v) with Turk solution (0.2% violet crystal in 30% acetic acid). The purity of the isolated cell populations was determined by the Panotic staining of cytospin preparations and by flow cytometry analysis (FACScan). The mean purity was 99% for the PBMC preparation. The number of cells was adjusted to 2 × 10^5^ or 1 × 10^6^ depending on the number of cells necessary for each experiment^23^.

### Cell viability assay by flow cytometry (BD™ cell viability kit)

Following the method described by Pires et al.^23^, PBMCs (1 × 10^6^ cells/well) were suspended in RPMI assay medium and incubated, in duplicate, in 96-well plates with culture medium—RPMI (negative control) or CVX (5 and 10 µg/mL; experimental group) for 1, 12, 24, 48, 72 and 96 h at 37 °C, in a humidified atmosphere of 5% CO_2_. Then that, 0.1 μL of propidium iodide (PI) and 0.2 μL of thiazole orange (TO) was added to each sample, stirred by vortexing, and then incubated for 5 min at room temperature. Acquisition and analysis were then performed in FACScan. The results are expressed in percentages.

### Cell proliferation assay by flow cytometry

Cell proliferation was evaluated using carboxyfluorescein diacetate succinimidyl ester (CFSE) as described by Pires et al.^23^. In brief, isolated PBMCs were stained with CFSE and plated in 96-well flat-bottom tissue culture plates at a concentration of 1 × 10^6^ cells/well containing 100 µL of RPMI assay medium. After that, PBMCs were incubated with RPMI (negative control), phytohemagglutinin—PHA (5 µg/mL; positive control), or CVX (5 and 10 µg/mL; experimental group) for 72 h at 37 °C, under an atmosphere of 5% CO_2_. After incubation, cell surface labeling was performed with monoclonal antibody PE anti-human CD3 and PerCP anti-human CD14, incubated for 30 min on ice and protected from light. PBMCs were washed with PBS to remove unbound antibodies. Acquisition and analysis were performed in FACScan. Cell proliferation is observed by the presence of decreasing histogram peaks on the left in the green region of the graph.

### Determination of reactive oxygen species (ROS)

A suspension of PBMCs (2 × 10^5^) obtained by isolation and resuspended in Hanks solution (1.26 mM CaCl_2_; 5.33 mM KCl; 044 mM KH_2_PO_4_; 0.50 mM MgCl_2_; 138 mM NaCl; NaHCO_3_ 4.0 nM; 0.30 mM Na_2_HPO_4_; 5.60 mM C_6_H_12_O_6_) were plated on black plates for fluorescence quantification. PBMCs were then incubated at 37 °C in a humid atmosphere containing 5% CO_2_ with Hanks solution (negative control), PMA 500 ng/mL (positive control group), or CVX (5 and 10 μg/mL; experimental group). After 30 min, 1, 2 and 3 h of incubation, 100 μL of a solution containing the DCFDA fluorescent probe (10 μM) diluted in Hanks solution were added followed by incubation for 30 min at 37 °C in the dark. The reading was performed with 485 nm and 528 nm excitation in a spectrophotometer (Synergy HT, Biotek). Data were expressed as absorbance^23^.

### Measurement of intracellular ROS levels

A peroxide-sensitive fluorescent probe 2′,7′-dichlorodihydrofluorescein diacetate (DCFDA) was used to measure intracellular levels of ROS. DCFDA is converted by intracellular esterases to 2′,7′-dichlorodihydrofluorescin, which is then oxidized by H_2_O_2_ to the highly fluorescent 2′,7′-dichloro-dihydro fluorescein (DCF). For this assay, 2 × 10^5^PBMCs were resuspended in RPMI without phenol red, and the cells were incubated with RPMI without phenol red (negative control), PMA (500 ng/mL; positive control), or CVX (5 and 10 µg/mL). After 3 h of incubation, 100 μL of DCFDA, diluted with 10 μM of PBS, was added and incubated for 30 min at 37 °C, under constant dark conditions. After incubation, cell surface labeling was performed with monoclonal antibody PerCP anti-human CD3 or PE anti-human CD14, incubated for 30 min on ice and protected from light. PBMCs were washed with PBS to remove unbound antibodies^23^. Acquisition and analysis were performed in FACScan. The results are expressed in percentages.

### Immunofluorescence

For immunofluorescence microscopy, 2 × 10^5^ isolated and stimulated human PBMCs for 3 h, as mentioned above were seeded on 70% alcohol-washed coverslips and treated with Poly-L-Lysine (Sigma Aldrich) and placed in 24-well plates. The cells were fixed with 4% paraformaldehyde at room temperature for 15 min. Next, cells were incubated with the MitoSOX™ Red Mitochondrial Superoxide Indicator (Thermo Fisher) for 30 min according to the manufacturer's instructions. After staining, the coverslips were mounted with Fluoroshield containing DAPI (Sigma Aldrich) and analyzed under a Nikon Eclipse 80i microscope with a 100× magnification oil immersion objective. The images were collected using constant automatic gain among the samples to quantify the differences in absolute levels of fluorescence intensity in different conditions. 50 fields of view of each condition were collected impartially. The acquired images were subsequently analyzed using ImageJ software (National Institutes of Health) to quantify the absolute total fluorescence intensity. The calculated fluorescence intensity of the fields of view was plotted as mean normalized intensity for the total number of cells^53^.

### Nitric oxide (NO) production assay

NO production by PBMCs incubated with and without CVX was determined in the supernatant. PBMCs, at a density of 2 × 10^5^/250 µL in RPMI assay medium, were plated in 96-well plates and incubated with RPMI (negative control), PMA (500 ng/mL; positive control) or CVX (5 and 10 µg/mL; experimental group) for 24, 48 e 72 h at 37 °C, in a humidified atmosphere of 5% CO_2_. After incubation, the cells supernatants were transferred to a reading plate, and Griess reagent was added (in the ratio of 1:1, v/v) for nitrite determination^54^. The absorbance was measured spectrophotometrically (Bio-Tek Synergy HT Multi-Detection, Winooski, VT), at 550 nm, data were compared to a standard curve prepared with NaNO_2_ (2.5 to 80 μM) and expressed in terms of µM nitrite released by 2 × 10^5^ cells.

### EIA cytokines measurements

For this assay, 2 × 10^5^ PBMCs resuspended in RPMI assay medium were plated and incubated with RPMI (negative control), Con-A (5 µg/mL; positive control), LPS (1 µg/mL; positive control), or CVX (5 and 10 µg/mL) at 37 °C in a humidified atmosphere (5% CO_2_) for 12 and 24 h. After centrifugation, the collected supernatant was used for the quantification of IL-2, IL-10 and IL-1β levels by specific enzyme immunoassay (EIA) according to manufacture instructions (BD OptEIA Human ELISA Set). The results were expressed in pg/mL of each cytokine.

### Cell viability—MTT assay

Mitochondrial activity was measured to assess cell viability according to Pires et al.^23^. In summary, PBMCs (2 × 10^5^ cells/well) were suspended in an RPMI (control), laminarin (a β-glucan receptor ligand; 100 µg/mL)^55^, N-tosyl-L phenylalanine chloromethyl ketone (an inhibitor of NF-κB Activation by Blocking Specific Cysteine Residues of IκB Kinase; NF-κB inhibitor; 30 µM)^24^, Ac-YVAD-cmk (a selective, irreversible inhibitor of interleukin-1β converting enzyme ICE; caspase-1 inhibitor; 50 µM)^25^, MCC950 (a selective NOD-like receptor protein-3-NLRP3 inhibitor; 10 µM)^26^, Apocynin (NADPH Oxidase ROS; 300 µM)^56^ and Rotenone (mROS inhibitor; 10 µM)^57^, diluted in RPMI medium, for 12 h at 37 °C, in a humidified atmosphere of 5% CO_2_. The concentration of the inhibitors used in this study was based on the literature as effective. Next, 10 μL of MTT (5 mg/mL) was added and incubated for 2 h. After centrifugation at 400×*g* for 5 min, the supernatant was removed and 100 μL of DMSO was added to dissolve the formed crystals. Subsequently, the plates were kept for 15 min at room temperature and evaluated in a spectrophotometer at 540 nm. The results were expressed in percentage compared to the respective controls.

### Pharmacological treatment with laminarin

To evaluate the Dectin, a β-glucan receptor, on CVX action on PBMCs, the cells were treated with laminarin (a β-glucan receptor ligand; 100 µg/mL) dissolved in RPMI, for 30 min^54^. Hereafter, the cells were incubated with CVX at a concentration of 10 µg/mL and the supernatant of this incubation used for the IL-10 quantification as described above. The concentration of the ligand used in this study was based on the literature as effective and that it did not cause an adverse effect on cell viability during the assay. Control cells (PBMCs incubated with culture medium) were incubated with the same concentration as the vehicle used to dissolve the ligand (laminarin)^58^.

### Pharmacological treatment with N-tosyl-l-phenylalanine chloromethyl ketone (NF-κB inhibitor), Ac-YVAD- cmk (caspase-1 inhibitor) and MCC950 (NLRP3 inhibitor)

**Figure 10:**
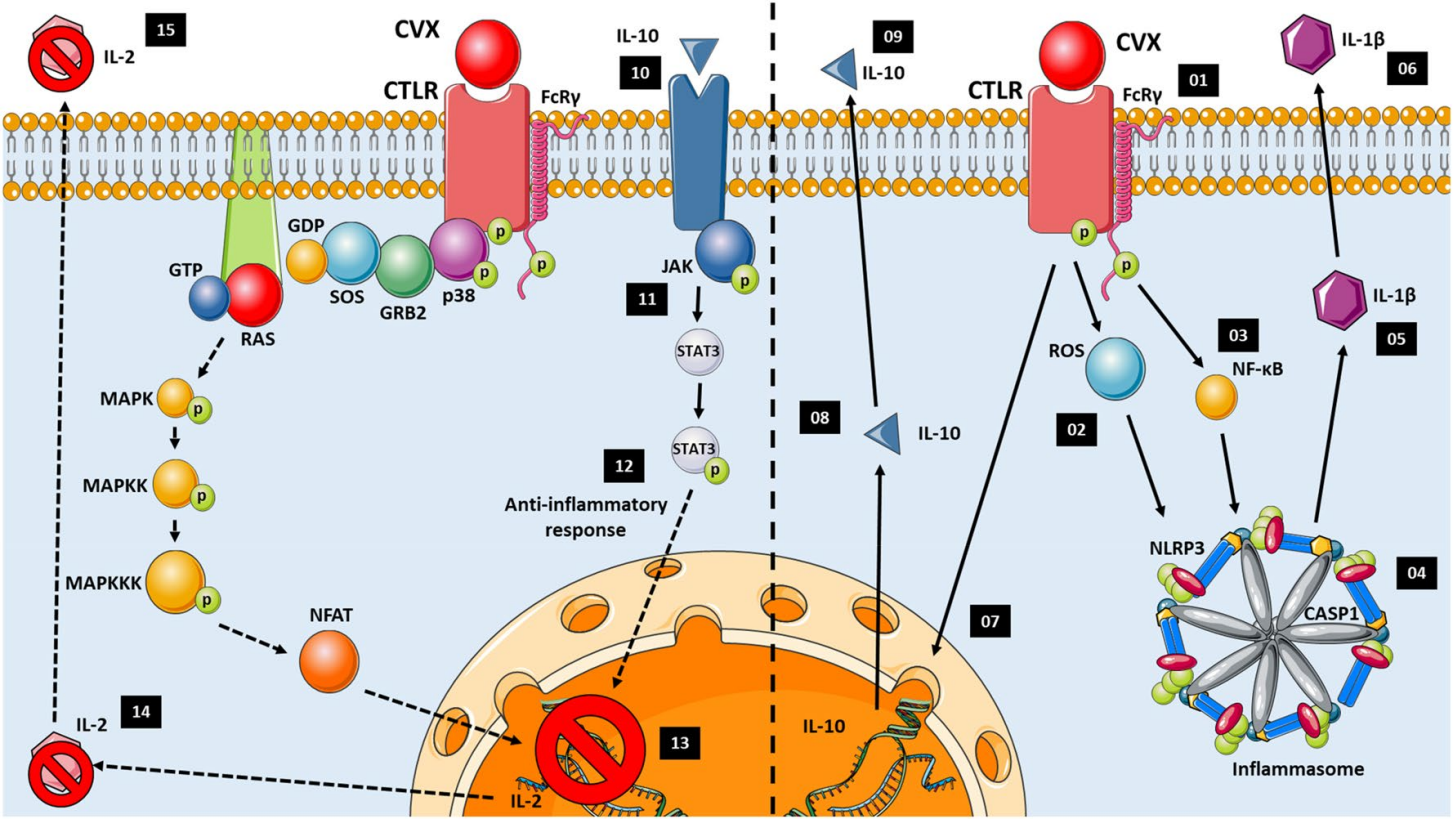
The mechanism of the CVX interaction with the CTLR proposed (Dectin) in the present study. The interaction of CVX with CTLR (1) induces ROS production (2) and NF-κB activation (3). ROS induce the activation of the NLRP3 inflammasome complex (4); stimulating the production (5) and release of the pro-inflammatory cytokine IL-1β (6). The IL-1β production by the interaction of CVX and CTLR, induces the transcription of IL-10 (7); and consequently, its release (8). The interaction of IL-10 with its specific receptor (9), induces JAK-1 phosphorylation (10), initiating signaling via STAT3 (11,12), performing an anti-inflammatory activity, inhibiting transcription (13), production (14) and IL-2 release (15).

To evaluate the participation of NF-κB and the inflammasome complex activation on the CVX action on PBMCs, the cells were treated with N-tosyl-l-phenylalanine chloromethyl ketone (TPCK) (30 µM), Ac-YVAD-cmk (50 µM), MCC950 (10 µM) dissolved in RPMI, for 30 min. The concentration of the inhibitors used in this study was based on the literature as effective and that it did not cause an adverse effect on cell viability during the assay. Subsequently, the cells were incubated with CVX at a concentration of 10 µg/mL and the supernatant collected and used for the IL-1β quantification (Fig. 10).

### Pharmacological treatment with apocynin (NADPH Oxidase inhibitor) and rotenone (mROS inhibitor)

To analyze the involvement of ROS through NADPH Oxidase and mitochondrial in signaling for inflammasome activation on CVX action on PBMCs, the cells were treated for 30 min with Apocynin (300 M) and Rotenone (10 M) dissolved in RPMI. The inhibitor concentration employed in this investigation was based on the literature and had no negative impact on viability during the experiment. Following that, the cells were treated with CVX at a concentration of 10 g/mL, and the supernatant was collected and utilized to quantify IL-1β.

### Immunoblotting

PBMCs resuspended in RPMI assay medium were plated and incubated with RPMI (negative control), LPS (1 µg/mL; positive control) or CVX (5 and 10 µg/mL) at 37 °C in a humidified atmosphere (5% CO_2_) for 3 h. The cells were lysed and homogenized with RIPA buffer containing phosphatase and protease inhibitors [50 mM Tris–HCl pH 7.4, 1% Triton X-100, 0.25% sodium deoxycholate, 150 mM NaCl, 1 mM ethylenediaminetetraacetic acid (EDTA), 1 mM PMSF, 5 μg/ml aprotinin, 5 μg/ml leupeptin, 1 mM Na_3_VO_4_]. Immunoblotting was performed using monoclonal antibodies anti-β-actin, anti-caspase-1 and anti-NLRP3. For β-actin, caspase-1 and NLRP3 determinations, total protein extracts were prepared, resolved by 10% SDS/PAGE and transferred onto a PVDF membrane (Hybond, Amersham Pharmacia Biotech) (Fig. S1). Immunoblotting was performed using monoclonal antibodies to the referent proteins. Blots were developed with 3,30-diaminobenzidine and hydrogen peroxide. The relative immunoreactivity bands of three independent experiments were quantified by densitometry using Image Studio Lite Ver 5.2 (LI-COR, Lincoln, Nebraska, EUA). The mean densitometry values of tested proteins were divided by the mean densitometry values of respective β-actin values to show the relative expression of each protein as a ration means of the protein/β-actin^53^.

### Molecular modeling approaches

The molecular dockings performed in this study were conducted on the ClusPro web server under the balanced coefficient^59^. The PDB codes for the structures used in the simulations were the following: 1uos (CVX); 3wh2 (CLEC4E); 5vyb (Dectin-2). All the images and interaction maps were made with UCSF Chimera and LIGPLOT+^60,61^.

The complexes obtained from dockings were assessed through molecular dynamics simulations using GROMACS 2018.1^62^ employing the CHARMM36-mar2019 force field^63^. All simulations were carried in a neutral net charge box with 5 Å from the farthest atom, solvated with TIP3P water and equilibrated with 100 mM of NaCl. The system was minimized with the Steepest Descent algorithm until reaching energy below 100 kJ/mol/nm. Then, an NVT ensemble was executed, generating velocities according to Maxwell–Boltzmann distribution at 300 K using the V-rescale thermostat^64^ followed by the NPT ensemble using the Beredsen barostat at 1bar^65^. Subsequently, a 10 ns step was executed using the Nose–Hoover Thermostat^66^ and Parrinello-Rahman barostat^67^. Non-bonded interactions were calculated within a radius of 12 Å using a switching function between 10 and 12 Å. Afterwards, the trajectories were analyzed and RMSD measures were extracted from the main interacting parties for stability assessment.

Further, the trajectories were subjected to a clusterization using the gromos method^68^ with a RMSD distribution of 3 Å. The central structures found in the 10 most populated clusters were subjected to a binding affinity prediction using the PRODIGY web server^69^.

### Statistical analysis

The means and S.E.M. of all data were obtained and compared by one-way ANOVA, followed by Tukey test with significance probability levels less than 0.05.

## Supplementary Information

Supplementary Figure S1.

## References

[1] Sano-Martins IS (2001). Coagulopathy following lethal and non-lethal envenoming of humans by the South American rattlesnake (*Crotalus durissus*) in Brazil. QJM.

[2] Saravia P (2002). Geographic and ontogenic variability in the venom of the neotropical rattlesnake *Crotalus durissus*: Pathophysiological and therapeutic implications. Rev. Biol. Trop..

[3] de Carvalho LH (2019). Local and systemic effects caused by *Crotalus durissus terrificus*, *Crotalus durissus collilineatus*, and *Crotalus durissus cascavella* snake venoms in swiss mice. Rev. Soc. Bras. Med. Trop..

[4] Rolim-Rosa, R., Vieira, E. G. J., Sillesvillarroel, M., Siracusa, Y. Q. & Iizuka, H. Análise comparativa entre os diferentes esquemas de hiperimunização empregados na produção de soros antiofídicos pelo Instituto Butantan (1957–1979). In *Mem. Inst. Butantan* 259–270 (1980).

[5] Schaeffer RC, Randall H, Resk J, Carlson RW (1988). Enzyme-linked immunosorbent assay (ELISA) of size selected crotalic venom antigens by Wyeth’s polyvalent antivenom. Toxicon.

[6] Da Silva RJ, Fecchio D, Barraviera B (1996). Antitumor effect of snake venoms. J. Venom. Anim. Toxins.

[7] Cardoso DF, Mota I (1997). Effect of *Crotalus* venom on the humoral and cellular immune response. Toxicon.

[8] Picolo G, Giorgi R, Cury Y (2000). delta-opioid receptors and nitric oxide mediate the analgesic effect of *Crotalus durissus terrificus* snake venom. Eur. J. Pharmacol..

[9] Rangel-Santos A, Lima C, Lopes-Ferreira M, Cardoso DF (2004). Immunosuppresive role of principal toxin (crotoxin) of *Crotalus durissus terrificus* venom. Toxicon.

[10] Sartim MA, Menaldo DL, Sampaio SV (2018). Immunotherapeutic potential of Crotoxin: Anti-inflammatory and immunosuppressive properties. J. Venom. Anim. Toxins Incl. Trop. Dis..

[11] Francischetti IM (1997). Convulxin, a potent platelet-aggregating protein from *Crotalus durissus* terrificus venom, specifically binds to platelets. Toxicon.

[12] Clemetson, K. J. & Clemetson, J. M. Platelet receptors. In *Platelets* 169–194 (Elsevier, 2013). 10.1016/B978-0-12-387837-3.00009-2.

[13] Prado-Franceschi J, Vital-Brazil O (1981). Convulxin, a new toxin from the venom of the South American rattlesnake *Crotalus durissus terrificus*. Toxicon.

[14] Murakami MT, Zela SP, Gava LM, Michelan-Duarte S, Cintra AC, Arni RK (2003). Crystal structure of the platelet activator convulxin, a disulfide-linked alpha4beta4 cyclic tetramer from the venom of *Crotalus durissus terrificus*. Biochem Biophys Res Commun..

[15] Batuwangala T, Leduc M, Gibbins JM, Bon C, Jones EY (2004). Structure of the snake-venom toxin convulxin. Acta Cryst..

[16] Drickamer K (1993). Evolution of Ca^2+^ dependent animal lectins. Prog. Nucl. Acid Res. Mol. Biol..

[17] Leduc M, Bon C (1998). Cloning of subunits of convulxin, a collagen-like platelet-aggregating protein from *Crotalus durissus terrificus* venom. Biochem. J..

[18] Hirotsu S, Mizuno H, Fukuda K, Qi MC, Matsui T, Hamako J, Morita T, Titani K (2001). Crystal structure of bitiscetin, a von Willebrand factor-dependent platelet aggregation inducer. Biochemistry.

[19] Sen U, Vasudevan S, Subbarao G, Mcclintock RA, Celikel R, Ruggeri ZM, Varughese KI (2001). Crystal structure of the von Willebrand factor modulator botrocetin. Biochemistry.

[20] Kanneganti T-D (2015). The inflammasome: Firing up innate immunity. Immunol. Rev..

[21] Sharma D, Kanneganti T-D (2016). The cell biology of inflammasomes: Mechanisms of inflammasome activation and regulation. J. Cell Biol..

[22] Latz E, Xiao TS, Stutz A (2013). Activation and regulation of the inflammasomes. Nat. Rev. Immunol..

[23] Pires WL (2017). Effect of BjcuL, a lectin isolated from *Bothrops jararacussu*, on human peripheral blood mononuclear cells. Toxicol. In Vitro.

[24] Ha KH, Byun MS, Choi J, Jeong J, Lee KJ, Jue DM (2009). N-tosyl-l-phenylalanine chloromethyl ketone inhibits NF-kappaB activation by blocking specific cysteine residues of IkappaB kinase beta and p65/RelA. Biochemistry.

[25] Xing Y, Cao R, Hu HM (2016). TLR and NLRP3 inflammasome-dependent innate immune responses to tumor-derived autophagosomes (DRibbles). Cell Death Dis..

[26] Tapia-Abellán A, Angosto-Bazarra D, Martínez-Banaclocha H, de Torre-Minguela C, Cerón-Carrasco JP, Pérez-Sánchez H, Arostegui JI, Pelegrin P (2019). MCC950 closes the active conformation of NLRP3 to an inactive state. Nat. Chem. Biol..

[27] Couper KN, Blount DG, Riley EM (2008). IL-10: The master regulator of immunity to infection. J. Immunol..

[28] Furukawa A (2013). Structural analysis for glycolipid recognition by the C-type lectins Mincle and MCL. Proc. Natl. Acad. Sci. USA..

[29] Clemetson JM, Polgar J, Magnenat E, Wells TN, Clemetson KJ (1999). The platelet collagen receptor glycoprotein VI is a member of the immunoglobulin superfamily closely related to FcalphaR and the natural killer receptors. J. Biol. Chem..

[30] Plato A, Willment JA, Brown GD (2013). C-type lectin-like receptors of the dectin-1 cluster: Ligands and signaling pathways. Int. Rev. Immunol..

[31] Carvalho EVMM, Oliveira WF, Coelho LCBB, Correia MTS (2018). Lectins as mitosis stimulating factors: Briefly reviewed. Life Sci..

[32] Shih C-H, Chiang T-B, Wang W-J (2017). Convulxin, a C-type lectin-like protein, inhibits HCASMCs functions via WAD-motif/integrin-αv interaction and NF-κB-independent gene suppression of GRO and IL-8. Exp. Cell Res..

[33] Yates A, Bergmann C, Van Hemmen JL, Stark J, Callard R (2000). Cytokine-modulated regulation of helper T cell populations. J. Theor. Biol..

[34] de Waal Malefyt R, Abrams J, Bennett B, Figdor CG, de Vries JE (1991). Interleukin 10(IL-10) inhibits cytokine synthesis by human monocytes: An autoregulatory role of IL-10 produced by monocytes. J. Exp. Med..

[35] Moore KW, O’Garra A, de Waal Malefyt R, Vieira P, Mosmann TR (1993). Interleukin-10. Annu. Rev. Immunol..

[36] Rhodes KA, Andrew EM, Newton DJ, Tramonti D, Carding SR (2008). A subset of IL-10-producing gammadelta T cells protect the liver from Listeria-elicited, CD8(+) T cell-mediated injury. Eur. J. Immunol..

[37] Sabat R (2010). IL-10 family of cytokines. Cytokine Growth Factor Rev..

[38] Sabat R (2010). Biology of interleukin-10. Cytokine Growth Factor Rev..

[39] Riley JK, Takeda K, Akira S, Schreiber RD (1999). Interleukin-10 receptor signaling through the JAK-STAT pathway. J. Biol. Chem..

[40] Vespa GN, Cunha FQ, Silva JS (1994). Nitric oxide is involved in control of *Trypanosoma cruzi*-induced parasitemia and directly kills the parasite in vitro. Infect. Immun..

[41] Macphail SE (2003). Nitric oxide regulation of human peripheral blood mononuclear cells: Critical time dependence and selectivity for cytokine versus chemokine expression. J. Immunol..

[42] Wilkins-Rodríguez AA, Escalona-Montaño AR, Aguirre-García M, Becker I, Gutiérrez-Kobeh L (2010). Regulation of the expression of nitric oxide synthase by *Leishmania mexicana* amastigotes in murine dendritic cells. Exp. Parasitol..

[43] Zhou R, Yazdi AS, Menu P, Tschopp J (2011). A role for mitochondria in NLRP3 inflammasome activation. Nature.

[44] Yang Y, Wang H, Kouadir M, Song H, Shi F (2019). Recent advances in the mechanisms of NLRP3 inflammasome activation and its inhibitors. Cell Death Dis..

[45] Pontes AS (2016). p38 MAPK is involved in human neutrophil chemotaxis induced by l-amino acid oxidase from *Calloselasma rhodosthoma*. Toxicon.

[46] Mills EL (2016). Succinate dehydrogenase supports metabolic repurposing of mitochondria to drive inflammatory macrophages. Cell.

[47] Liu T, Zhang L, Joo D, Sun SC (2017). NF-κB signaling in inflammation. Signal Transduct Target Ther..

[48] Kerscher B, Willment JA, Brown GD (2013). The Dectin-2 family of C-type lectin-like receptors: An update. Int. Immunol..

[49] Feinberg H (2017). Mechanism of pathogen recognition by human dectin-2. J. Biol. Chem..

[50] Chiffoleau E (2018). C-type lectin-like receptors as emerging orchestrators of sterile inflammation represent potential therapeutic targets. Front. Immunol..

[51] Nagae M (2014). A platform of C-type lectin-like receptor CLEC-2 for binding O-glycosylated podoplanin and nonglycosylated rhodocytin. Structure.

[52] Pinto TGA, Kaneko TM, Pinto AF (2015). Controle biológico de qualidade de produtos farmacêuticos, correlatos e cosméticos.

[53] Paloschi MV (2020). Cytosolic phospholipase A2-α participates in lipid body formation and PGE2 release in human neutrophils stimulated with an l-amino acid oxidase from *Calloselasma rhodostoma* venom. Sci. Rep..

[54] Ding AH, Nathan CF, Stuehr DJ (1988). Release of reactive nitrogen intermediates and reactive oxygen intermediates from mouse peritoneal macrophages. Comparison of activating cytokines and evidence for independent production. J. Immunol..

[55] Giaimis J (1993). Both mannose and beta-glucan receptors are involved in phagocytosis of unopsonized, heat-killed *Saccharomyces cerevisiae* by murine macrophages. J. Leukoc. Biol..

[56] Stolk J, Hiltermann TJ, Dijkman JH, Verhoeven AJ (1994). Characteristics of the inhibition of NADPH oxidase activation in neutrophils by apocynin, a methoxy-substituted catechol. Am. J. Respir. Cell Mol. Biol..

[57] Handa O (2004). Tumor necrosis factor-alpha-induced cytokine-induced neutrophil chemoattractant-1 (CINC-1) production by rat gastric epithelial cells: Role of reactive oxygen species and nuclear factor-kappaB. J. Pharmacol. Exp. Ther..

[58] Ferreira KS, Bastos KR, Russo M, Almeida SR (2007). Interaction between *Paracoccidioides brasiliensis* and pulmonary dendritic cells induces interleukin-10 production and toll-like receptor–2 expression: Possible mechanisms of susceptibility. J. Infect. Dis..

[59] Kozakov D (2017). The ClusPro web server for protein–protein docking. Nat. Protoc..

[60] Pettersen EF (2004). UCSF Chimera?A visualization system for exploratory research and analysis. J. Comput. Chem..

[61] Wallace AC, Laskowski RA, Thornton JM (1995). LIGPLOT: A program to generate schematic diagrams of protein-ligand interactions. Protein Eng..

[62] Abraham MJ (2015). GROMACS: High performance molecular simulations through multi-level parallelism from laptops to supercomputers. SoftwareX.

[63] Huang J (2017). CHARMM36m: An improved force field for folded and intrinsically disordered proteins. Nat. Methods.

[64] Bussi G, Donadio D, Parrinello M (2007). Canonical sampling through velocity rescaling. J. Chem. Phys..

[65] Berendsen HJC, Postma JPM, van Gunsteren WF, DiNola A, Haak JR (1984). Molecular dynamics with coupling to an external bath. J. Chem. Phys..

[66] Hoover WG (1985). Canonical dynamics: Equilibrium phase-space distributions. Phys. Rev. A.

[67] Parrinello M, Rahman A (1981). Polymorphic transitions in single crystals: A new molecular dynamics method. J. Appl. Phys..

[68] Daura X, van Gunsteren WF, Mark AE (1999). Folding-unfolding thermodynamics of a β-heptapeptide from equilibrium simulations. Proteins Struct. Funct. Genet..

[69] Xue LC, Rodrigues JP, Kastritis PL, Bonvin AM, Vangone A (2016). PRODIGY: A web server for predicting the binding affinity of protein–protein complexes. Bioinformatics.

