# Supplementary material for: Inflammasome NLRP3 activation induced by Convulxin, a C-type lectin-like isolated from Crotalus durissus terrificus snake venom
Source: Sci Rep. 2022 Mar 18;12:4706. doi: 10.1038/s41598-022-08735-7 (PMC8933474; doi:10.1038/s41598-022-08735-7)
Supplement: Supplementary file 1 — Supplementary Figure S1. [file 41598_2022_8735_MOESM1_ESM.pptx]

## Slide 1
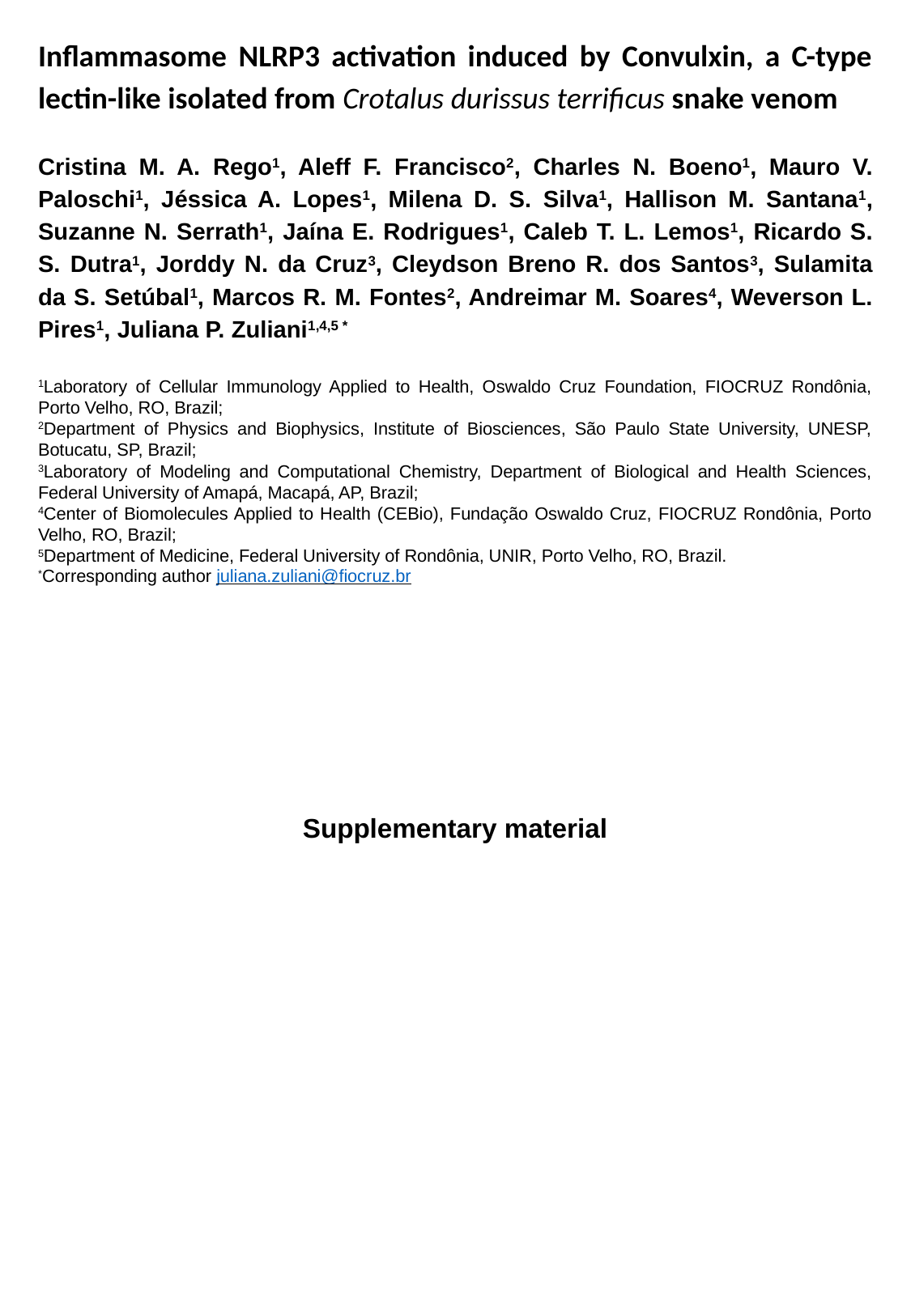

Inflammasome NLRP3 activation induced by Convulxin, a C-type lectin-like isolated from Crotalus durissus terrificus snake venom
Cristina M. A. Rego1, Aleff F. Francisco2, Charles N. Boeno1, Mauro V. Paloschi1, Jéssica A. Lopes1, Milena D. S. Silva1, Hallison M. Santana1, Suzanne N. Serrath1, Jaína E. Rodrigues1, Caleb T. L. Lemos1, Ricardo S. S. Dutra1, Jorddy N. da Cruz3, Cleydson Breno R. dos Santos3, Sulamita da S. Setúbal1, Marcos R. M. Fontes2, Andreimar M. Soares4, Weverson L. Pires1, Juliana P. Zuliani1,4,5 *
1Laboratory of Cellular Immunology Applied to Health, Oswaldo Cruz Foundation, FIOCRUZ Rondônia, Porto Velho, RO, Brazil;
2Department of Physics and Biophysics, Institute of Biosciences, São Paulo State University, UNESP, Botucatu, SP, Brazil;
3Laboratory of Modeling and Computational Chemistry, Department of Biological and Health Sciences, Federal University of Amapá, Macapá, AP, Brazil;
4Center of Biomolecules Applied to Health (CEBio), Fundação Oswaldo Cruz, FIOCRUZ Rondônia, Porto Velho, RO, Brazil;
5Department of Medicine, Federal University of Rondônia, UNIR, Porto Velho, RO, Brazil.
*Corresponding author juliana.zuliani@fiocruz.br
Supplementary material

## Slide 2
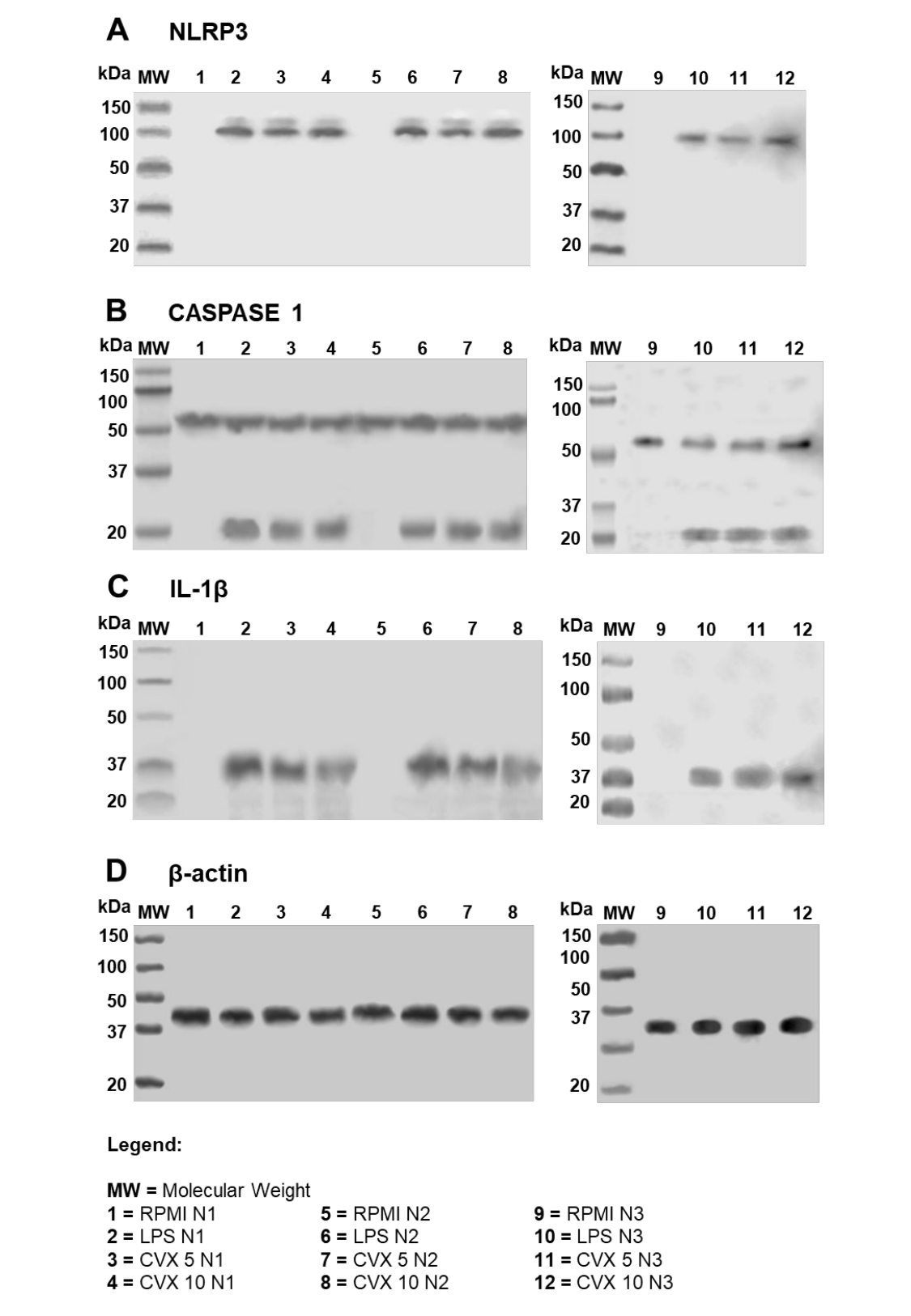

## Slide 3
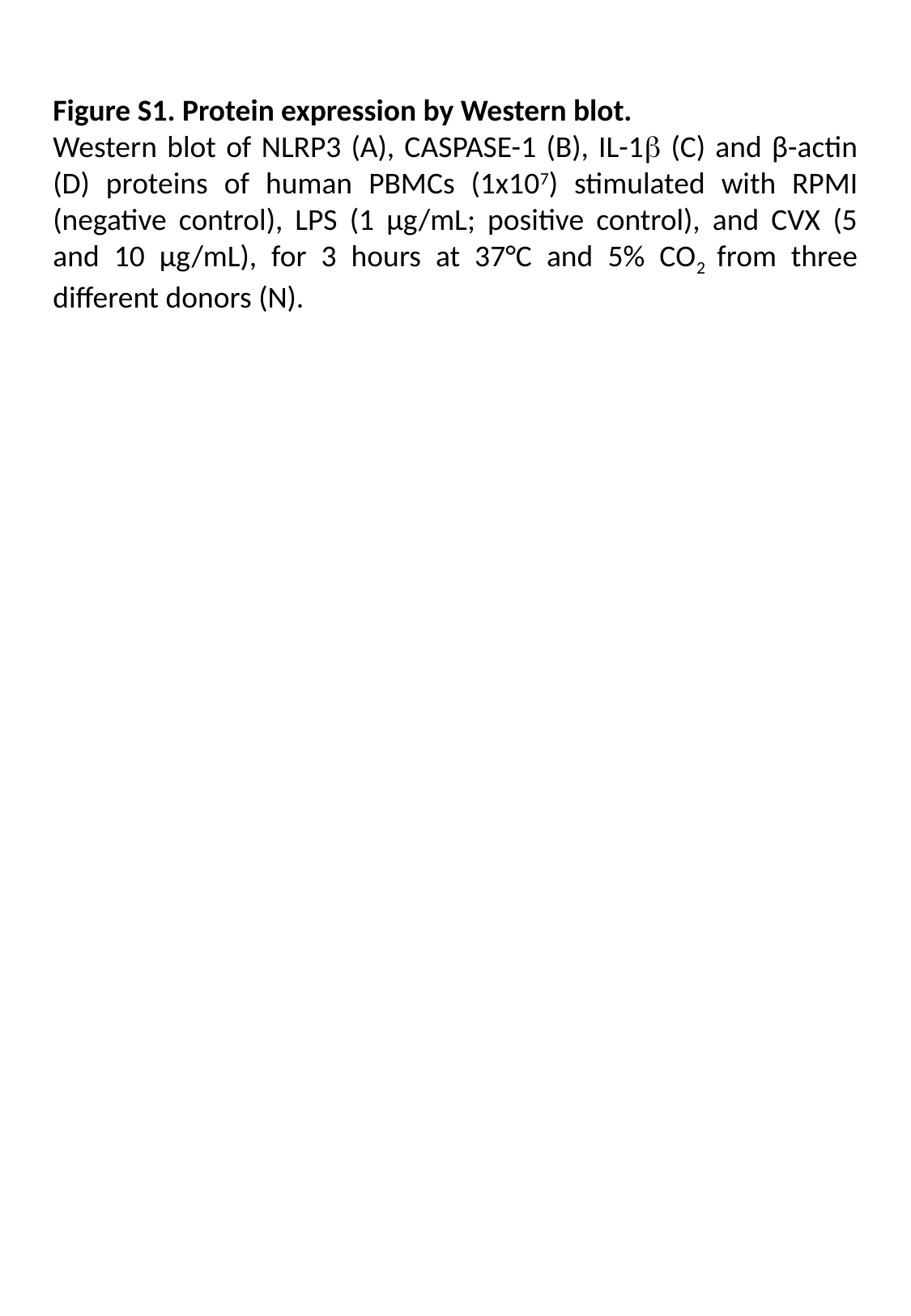

Figure S1. Protein expression by Western blot.
Western blot of NLRP3 (A), CASPASE-1 (B), IL-1b (C) and β-actin (D) proteins of human PBMCs (1x10^7^) stimulated with RPMI (negative control), LPS (1 μg/mL; positive control), and CVX (5 and 10 μg/mL), for 3 hours at 37°C and 5% CO2 from three different donors (N).
